# A Novel Method to Evaluate the Community Built Environment Using Photographs – Environmental Profile of a Community Health (EPOCH) Photo Neighbourhood Evaluation Tool

**DOI:** 10.1371/journal.pone.0110042

**Published:** 2014-11-04

**Authors:** Clara K. Chow, Daniel J. Corsi, Karen Lock, Manisha Madhavan, Pam Mackie, Wei Li, Sun Yi, Yang Wang, Sumathi Swaminathan, Patricio Lopez-Jaramillo, Diego Gomez-Arbelaez, Álvaro Avezum, Scott A. Lear, Gilles Dagenais, Koon Teo, Martin McKee, Salim Yusuf

**Affiliations:** 1 Population Health Research Institute, Hamilton Health Sciences, McMaster University, Hamilton, Ontario, Canada; 2 The George Institute for Global Health and Westmead Hospital Sydney Medical School, University of Sydney, Sydney, NSW, Australia; 3 European Centre on Health of Societies in Transition, London School of Hygiene and Tropical Medicine, London, United Kingdom; 4 Harvard Center for Population & Development Studies, Cambridge, MA, United States of America; 5 Cardiovascular Institute & Fuwai Hospital, Chinese Academy of Medical Sciences, Beijing, China; 6 St. John's Research Institute, St John's National Academy of Health Sciences, Bangalore, Karnataka, India; 7 Research Direction, Fundacion Oftalmologica de Santander-Clinica Carlos Arila Lulle and Medical School, Universidad de Santander, Bucaramanga, Colombia; 8 Dante Pazzanese Institute of Cardiology, São Paulo, Brazil; 9 Faculty of Health Sciences, Simon Fraser University and Division of Cardiology, Providence Health Care, Vancouver, B.C., Canada; 10 Institut universitaire de cardiologie et de pneumologie de Québec (Université Laval), Quebec City, Quebec, Canada; Arizona State University, United States of America

## Abstract

**Background:**

Previous research has shown that environments with features that encourage walking are associated with increased physical activity. Existing methods to assess the built environment using geographical information systems (GIS) data, direct audit or large surveys of the residents face constraints, such as data availability and comparability, when used to study communities in countries in diverse parts of the world. The aim of this study was to develop a method to evaluate features of the built environment of communities using a standard set of photos. In this report we describe the method of photo collection, photo analysis instrument development and inter-rater reliability of the instrument.

**Methods/Principal Findings:**

A minimum of 5 photos were taken per community in 86 communities in 5 countries according to a standard set of instructions from a designated central point of each community by researchers at each site. A standard pro forma derived from reviewing existing instruments to assess the built environment was developed and used to score the characteristics of each community. Photo sets from each community were assessed independently by three observers in the central research office according to the pro forma and the inter-rater reliability was compared by intra-class correlation (ICC). Overall 87% (53 of 60) items had an ICC of ≥0.70, 7% (4 of 60) had an ICC between 0.60 and 0.70 and 5% (3 of 60) items had an ICC ≤0.50.

**Conclusions/Significance:**

Analysis of photos using a standardized protocol as described in this study offers a means to obtain reliable and reproducible information on the built environment in communities in very diverse locations around the world. The collection of the photographic data required minimal training and the analysis demonstrated high reliability for the majority of items of interest.

## Introduction

Previous research has shown that environments with features that encourage walking are associated with increased physical activity, with potential to lower rates of obesity. Features of the physical environment that encourage walking include presence of walking paths, traffic lights, and cross-walks that reduce the risks from vehicular traffic; green space, the presence of aesthetic architecture, structures, natural features, mixed-land use and the cleanliness and maintenance of the environment. [1]

A number of instruments now exist to capture the physical environment of communities. The methods used by these can be broadly classified into 3 categories. The first include methods which use geocoded data within Geographical Information Systems (GIS) [2] to analyse relationships between individuals and places. The second uses questionnaires administered to residents of communities to collate information on individual perceptions of the community they live in. [3] The third involves systematic observation or audit, in which a trained individual conducts an assessment of a community by observing and recording information along a pre-specified route. [4] [5]

The three methods described collect complementary data on the physical environment, but the last of these, from systematic observation, is onerous and experience so far has largely been limited to urban environments in North America, Europe and Australia. [6] [7] Consequently, it is not clear how applicable they will be when used in other parts of the world. [8]

We have developed instruments to capture information about the community environment through direct observations (EPOCH1) and surveys of perceptions among individuals residing in communities (EPOCH2). [9], [10] The capture of digital photos of the built environment and off-line analysis of data presented a potentially efficient and complementary mode of environmental assessment. Uses of photos have been embraced to promote social change in community health initiatives, e.g. Photovoice [11] and there are a number of studies that have used new forms of geospatial imaging, such as Google Streets, to capture the health-related aspects of communities, [12] but the use of photos to measure a community's built environment is still in its infancy.

The items included in this instrument were drawn from our earlier review of the literature which we conducted at the time we developed the family of EPOCH instruments. [13] Across the family of EPOCH instruments we aimed to measure all the constructs that we identified as environmental factors actually or potentially associated with cardiovascular disease (CVD). The EPOCH photo instrument mainly captured measures of the neighbourhood environment with respect to walkability; these constructs are summarised in the second table of our previous review. [13] Our review drew on a broad range of literature from many disciplines and included a number of instruments. Those instruments that have been tested for reliability and validity were particularly influential in the development of our method. (**Table 1**) [4], [14]–[17]. Four of them use a direct assessment method. [16] The fifth, NEWS (Neighborhood Environment Walkability Scale), captures individuals' perceptions of the neighborhood in which they live; in terms of sidewalks, street connectivity, safety, surroundings, and overall satisfaction. Since most of the direct measure instruments need significant human resources for data collection, whereby a researcher is required to walk individual streets to assess traffic, design, connectivity, and accessibility, our aim was to develop a method that could be potentially applicable worldwide and is less onerous with respect to data collection, thereby enabling a rapid and efficient assessment of a community environment for walkability.

**Table 1:** Existing published and validated instruments that examine the physical environment of communities compared to the current instrument.

Instrument	Measurement type	Where instrument has been tested	Constructs measured
Systematic Pedestrian and Cycling Environment Scan (SPACES) (Pikora et al, Am J Prev Med 2002)	Direct measure – observers auditing neighbourhood (In the broader evaluation the audit data was supplemented with data from GIS and secondary sources)	Australia	‘**Functional**’ – foot paths, street width, curb type; **Pedestrian Safety** – crosswalks, lighting; marked lanes **Aesthetics** – trees, gardens, sights; **Destination facilities** – vehicle and bike parking; **Subjective assessment** – as judged by observer, attractiveness for walking, cycling
Irvine-Minnesota Inventory (Day et al, Am J Prev Med 2006)	Direct measure – observers auditing neighbourhood	USA	162 items organised into 4 domains – **accessibility** (e.g. land-use mix, density of destinations, places for exercise/activity, physical barriers and amenities for walking/cycling); **pleasurability**- e.g. attractive destinations, architectural character, street furnishings, public spaces; **safety from traffic** e.g. sidewalks, bike lanes, low speed limits, angled parking, crosswalks, ; **safety from crime** – graffiti, street maintenance, street lighting.
Built environment site survey checklist (BESSC) (Weich et al, Health & Place, 2001)	Direct measure – observers auditing neighbourhood	England	27 item checklist – **Characteristics of buildings** – type, height, age, access, **Space around buildings** - provision of gardens, public space/common gardens or spaces; **Facilities and accessibility** – to shops, public transport, GP, school, pub; **Safety and security** – derelict land, graffiti, neighbourhood watch signs, vandalism.
Walkability Index (Frank et al, Brit J Sports Med 2009)	Direct measure – GIS/secondary data-sources from parcel-based land use data, street centreline files, census data	USA	**Net residential density** – residential units to the land area devoted to residential use per block; **retail floor area ratio** – retail building area divided by retail land floor area; **intersection density**- connectivity of street network; **land use mix** – diversity of land use.
Neighborhood Walkability Scale (NEWS) (Cerin et al, Medicine & Science in Sports & Exercise 2006)	Perceived measure – surveys of resident's perceptions of the environmental attributes	USA	Residential density, proximity to stores and facilities, perceived access to these destinations, street connectivity, facilities for walking and cycling, aesthetics, safety from traffic and crime
EPOCH Photo Neighbourhood Evaluation Tool (EP-NET)	Observers in communities take photos of their communities; central based observers code them using the evaluation tool	International (Brazil, Canada, China Colombia, India)	**Pedestrian safety** – Traffic signals/signs, street width, median strip; sidewalks, cross walks, Bicycle lanes and quality (presence/absence and quality); **Aesthetics/Beatification** – natural features, man-made landscaping, building design/architecture, street furniture/public art; **Community disorder** – litter, garbage, graffiti, maintenance/derelict buildings; **Urban density** – street density, vehicle density; **Overall appeal** - Subjective assessment by central photo observer of overall appeal – safety, suitability for walking, attractiveness.

The aim of this study was to develop a method to evaluate features of the built environment of communities using a standard set of photos. In this report we describe the method of photo collection, the development of an instrument for analysis, the inter-rater reliability properties of the instrument, and the creation of summary scores. Given the limited geographical scope of many previously described built environment instruments, we also wanted to assess its applicability in a much wider set of countries.

## Methods

### Ethics statement

The EPOCH instruments were approved by the Hamilton Health Sciences/McMaster Health Sciences Research Ethics board which operates in compliance with the ICH Good Clinical Practice Guidelines and the Tri-Council Policy Statement: Ethical Conduct for Research Involving Humans and Division 5 Health Canada Food and Drug Regulations. No data from human participants was used in the current analyses.

### Setting

The study was conducted in a convenience sample of 86 urban and rural communities (**Table 2**) from 5 countries (Canada, Colombia, Brazil, China and India) that were involved in the Prospective Urban and Rural Epidemiology (PURE) study of cardiovascular risk factors and disease [18] and in which the Environmental Profile of a Community's Health (EPOCH) 1 and 2 tools were developed. [9], [10] Investigators were encouraged to include urban and rural communities from a range of socioeconomic areas that had a diverse range of physical environments.

**Table 2:** Communities studied.

Country	Urban	Rural	Total
Canada	24	13	37
Brazil	3	3	6
Colombia	8	6	14
China	10	4	14
India	5	10	15
Total	50	36	86

### Photos

**Figure 1:**
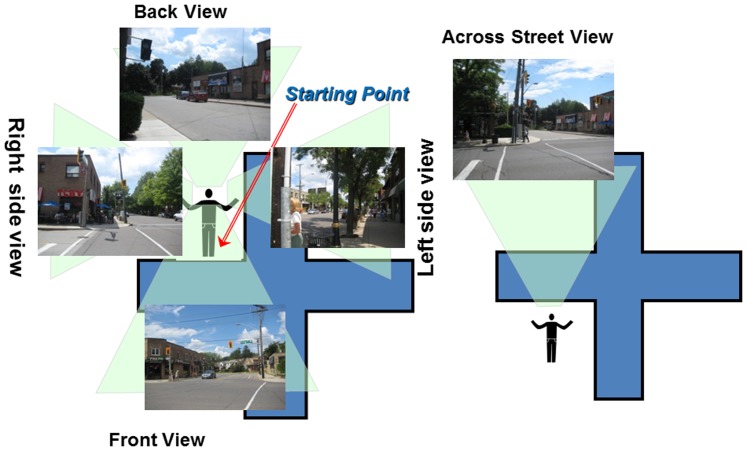
How photos were taken. This diagram shows how the photos were taken. The cross indicates an intersection. The individual must stand at their start point and take photos of each direction from their start points. (front, 2 sides, back). Then they go across from where they were standing to take a picture of their start point. All the photos must show clear view of the street and roads in the neighbourhood without any cars, buildings or pedestrians blocking the view.

Research assistants from each country were trained using a prepared set of slides and a manual explaining the procedures to be used. Photos were taken using a standardized protocol by researchers doing the ‘Community Observation Walk’ element of the EPOCH 1 assessment. [9] In brief the community observation walk took place in the commercial or central shopping district of the community and began at a central location. From this designated ‘start-point’ photos were taken to capture a 360 degree view of the community with a minimum of 5 photos. As illustrated in **Figure 1**, 4 of these photos were taken from the start point in each of 4 directions and the 5^th^ photo was of the start point, taken from across the street. From some communities we had more than this number of photos and in a few only 4 good quality photos could be assessed. Assessors were instructed as to where to stand to take photos, the views they were to obtain, and how to overlap images to achieve full coverage of a street scene. They were also given basic instructions on camera use, lighting and angle to take photos. In each community, 3 observers conducted the walk and we were sent 3 sets of photos from each community. However as these photos were found to be very similar when reviewed and our focus was on the reliability of external observers to audit the communities, we used only the first set of photos for the current analyses.

### Photo analysis

We scored each set of photos centrally using a standard form developed for this study. We identified the components to include in this form from reviewing literature on existing instruments as described above. The photos were assessed for ease of walking and biking, street type, pedestrian density, traffic, safety features such as the presence and quality of cross walks and median strips, aesthetics of the neighborhood (Appendix S1). The EPOCH Photo Neighbourhood Evaluation Tool (EP-NET) and accompanying manual was used by trained assessors to evaluate photos systematically. The first 32 items of the EP-NET instrument are objective measures, asking about the presence and absence of items and the number of items. Items 33 to 37 are items that ask the photo assessor to give an overall measure with respect to a construct. All items included in the instrument are listed in **Table 3**. All of the constructs included in the EP-NET instrument are drawn from the literature; however we adapted the exact means of measurement to be appropriate when applied to the medium of photography. For example a number of instruments have items that capture features of neighbourhood beautification. Our literature review indicated that the presence of grass/flowers/trees/public art/interesting features were consistent with the construct of neighbourhood beautification. These could have been captured by self-report by residents or by direct counting from neighbourhood audits. However in EP-NET we included the presence/absence of these items and a semi-quantitative measure of how many of these items were present e.g. none, 1 or 2, some or many. For items 33 to 37, the response scales were based on the NEWS questionnaire. [14]

**Table 3:** Features of communities and reliability of measures.

Questionnaire item	ICC	95% CI
Q1. Are sidewalks present (yes/no)	1.00	(1.00 , 1.00)
Q2. Type of sidewalk present in photos (complete, partial)	0.92	(0.89 , 0.95)
Q3. Concrete (yes/no)	0.92	(0.89 , 0.94)
Q3. Paving bricks (yes/no)	0.90	(0.87 , 0.93)
Q3. Asphalt (yes/no)	0.81	(0.75 , 0.87)
Q4. Quality of sidewalk (1 poor to 4 well maintained)	0.90	(0.86 , 0.93)
Q5. Bicycle lanes present (yes/no)	1.00	(1.00 , 1.00)
Q6. Quality of bicycle lanes (1 low to 3 high quality)	0.95	(0.93 , 0.97)
Q7. Grass/dirt strip present between sidewalk and road (no, some, all)	0.90	(0.86 , 0.93)
Q8. Parking lot present in photos (none, 1,2,≥3)	0.89	(0.85 , 0.93)
Q9. Width of street/road (1, 2/3, 4/5, >5 lanes)	0.88	(0.83 , 0.92)
Q10. Level of pedestrian density (1 low to 4 high)	0.93	(0.90 , 0.95)
Q11. Amount obstacles seen in photos (1 to 4)	0.78	(0.70 , 0.84)
Q12. Level of motor vehicle density (1 to 4)	0.89	(0.84 , 0.92)
Q13. Number of bicycles seen (continuous count)	0.87	(0.82 , 0.91)
Q13. Number of cars seen (continuous count)	0.92	(0.89 , 0.95)
Q13. Number of buses seen^1^	0.39	(0.25 , 0.52)
Q13. Number of rickshaws seen (continuous count)	0.85	(0.79 , 0.89)
Q13. Number of motorcycles/scooters seen^2^	0.67	(0.56 , 0.76)
Q13. Number of trucks seen	0.85	(0.80 , 0.90)
Q14. Amount of parked cars seen (1 none to >4 cars)	0.90	(0.86 , 0.93)
Q15. Are there crosswalks present (yes/no)	0.90	(0.85 , 0.93)
Q16. Number of crosswalks present (1/2, 3or 4, >4 crosswalks)	0.87	(0.82 , 0.91)
Q17a. White/coloured painted lines (yes/no)	0.89	(0.85 , 0.92)
Q17b. Different road surface or paving (yes/no)	0.89	(0.85 , 0.92)
Q17c. Traffic signals (yes/no)	0.75	(0.53 , 0.86)
Q17d. Stop/yield signs (yes/no)	0.79	(0.71 , 0.85)
Q17e. Pedestrian activated signal (yes/no)	0.94	(0.91 , 0.96)
Q17f. Pedestrian crossing signs (yes/no)	0.85	(0.79 , 0.89)
Q18. Median strip quality (1 no strip to 4 high quality strip)	0.95	(0.93 , 0.96)
Q19. Open field (yes/no)	0.76	(0.67 , 0.82)
Q19. Bodies of water (yes/no)	0.75	(0.66 , 0.82)
Q19. Mountains/hills (yes/no)^2^	0.74	(0.65 , 0.81)
Q19. Greenbelt/forest (yes/no)^1^	0.30	(0.12 , 0.47)
Q19. Desert (yes/no)	.	( . , .)
Q20. Percentage of natural feature present in photos	0.95	(0.93 , 0.97)
Q21. Number of trees planted (1 none to 4 many)	0.75	(0.65 , 0.82)
Q22. Number of man-made landscapes present (1 none to 4 many)	0.85	(0.79 , 0.90)
Q23. Graffiti present (1 none to 4 many)^2^	0.60	(0.48 , 0.70)
Q24. Litter/garbage present (1 none to 4 many)	0.84	(0.78 , 0.89)
Q25. Benches (yes/no)	0.84	(0.78 , 0.89)
Q25. Trashcan (yes/no)	0.86	(0.80 , 0.90)
Q25c. Newspaper boxes (yes/no)	0.79	(0.72 , 0.85)
Q25. Bike rack (yes/no)	0.83	(0.77 , 0.88)
Q25. Parking meter (yes/no)^2^	0.74	(0.66 , 0.82)
Q25. Street lamp (yes/no)	0.87	(0.82 , 0.91)
Q25. Bus shelter (yes/no)	0.83	(0.76 , 0.88)
Q25. Phone booth (yes/no)^2^	0.72	(0.63 , 0.80)
Q26. Number of public art displayed (1 none to 4 many)	0.81	(0.73 , 0.86)
Q27. Are buildings or houses present in photos (yes/no)	1.00	(1.00 , 1.00)
Q28. Number of buildings, houses, and/or structures present^1^	0.86	(0.80 , 0.90)
Q29. Amount of awnings present in photo (1 none to 4 many)	0.86	(0.81 , 0.90)
Q30. Number of derelict or vacant buildings and homes present (none, 1,2,≥3)	0.85	(0.80 , 0.90)
Q31. Evaluate exterior of structure and buildings and/or houses maintained (1 poor to 3 well maintained)	0.78	(0.70 , 0.84)
Q32. Diversity of buildings' and/or houses' design and architecture (1 minimal to 3 many)^2^	0.66	(0.55 , 0.75)
Q33. Opinion: The neighbourhood is very safe and pedestrian friendly (1 strongly disagree to 4 strongly agree)^2^	0.72	(0.62 , 0.80)
Q34. Opinion: The streets and sidewalks in neighbourhood are suitable for walking (1 to 4)^2^	0.74	(0.65 , 0.81)
Q35. Opinion: The streets and sidewalks in neighbourhood are suitable for biking (1 to 4)^1^	0.27	(0.14 , 0.41)
Q36. Opinion: The buildings, homes and structures in this neighbourhood are very (1 to 4^2^)	0.63	(0.52 , 0.72)
Q37. Opinion: The neighbourhood as a whole is aesthetically very appealing (1 to 4)^2^	0.74	(0.65 , 0.82)

Note: 1: Items with low reliability and 2: Items with moderate reliability. All other items had high reliability.

### Statistical analysis

We describe the characteristics of the communities overall and by sub-group (rural/urban and by country/regional grouping) using descriptive statistics. To assess the reliability of extraction of data from photos, each set of photos was scored independently by 3 individual observers based in Hamilton, Canada and trained using a study manual. The inter-rater reliability was assessed using the intra-class correlation coefficient (ICC) statistic, derived from a two-way random effects model. [9] The range of the ICC is between 0.0 and 1.0 and will be higher when less variation is present between observers. We defined a high ICC (greater than 0.75) to indicate good agreement and a low ICC (less than 0.4) to indicate poor agreement. All analyses were performed in Stata version 12.1.

## Results

### Descriptive characteristics

The three observers' responses on each characteristic are in detailed in Appendix S2. Observers reported the time taken to code a set of photos from a community to be on average 10 to 15 minutes and a maximum of 20 minutes per community. Observers reported higher scores for pedestrian facilities and safety characteristics in Canada compared to other countries and in urban compared to rural communities. For example, observer 1 noted 97.3% of communities in Canada had sidewalks, but this was 33.3% of communities in India. No cross-walks were reported in 10.8% of communities in Canada, but this was 86.7% of communities in India. Scores for beautification/aesthetic features were generally higher in Canada, though there was some variation. For example there were many planted trees in 32.4% of communities in Canada, but this was 15% in Brazil/Colombia, 6.7% in India and 42.9% in China. No man-made landscapes were observed to be present in 21.6% of communities in Canada compared to 60% in Brazil/Colombia, 93.3% of India and 21.4% of China. Scores for community disorder were higher in rural communities. Urban communities scored higher on overall appeal in all countries. (Appendix S2).

### Reliability

**Table 3** summarizes the inter-rater reliability for each community characteristic evaluated and **Table 4** summarizes the reliability measures by regions. The reliability of each item's measure by region is in the ‘Combined table’ in Appendix S2. Overall 77% (46 of 60) items had an ICC of ≥0.75, 17% (10 of 60) had an ICC between 0.60 and 0.74, 0% had an ICC between 0.4 and 0.59 and 5% (3 of 60) items had an ICC ≤0.40. One item, the presence of desert, could not be assessed for reliability as it did not occur in any of the communities included in the sample. Variables with a score of 1.0 were those exhibiting no variation, such as the presence of buildings (Q27), which was universal. The three items with low ICCs were: 1) the number of buses seen in the photos, where some assessors seemed to miss some of them; 2) the presence of greenbelt/forest, which highlighted differing interpretations of what constituted a greenbelt/forest and whether to include it if it was only in the distant background of the photo, and 3) the observer's opinion about suitability for biking, which again revealed different interpretations.

**Table 4:** Reliability by region. Number of items and percentage of all items with ICC in the following ranges, 60 items in total.

Group	No. of communities	ICC≥0.7	ICC 0.4–0.7	ICC<0.4
Overall	86	53/60 (88%)	4/60 (7%)	3/60 (5%)
Canada*	37	40/60 (67%)	13/60 (22%)	5/60 (8%)
Brazil/Columbia	20	48/60 (80%)	8/60 (13%)	4/60 (7%)
India/China	29	45/60 (75%)	9/60 (15%)	6/60 (10%)

*ICC not calculable for 2 items here due to too few counts.

### Creation of summary scores

To enable some qualitative examination of face validity, we have created a means of summarizing items into simple scores across 5 domains. For each domain, points were allocated to the main characteristics contributing to components of the domain and the total points summed within domains (**Table 5**). We described the community as high scoring if they had a score in the top tertile of scores and a low scoring community if they were in the bottom tertile of scores. The domains and their components were: 1) ***Pedestrian facilities and safety*** features – presence of sidewalks, completeness and quality of sidewalks, cross walks, and cross walk safety *features* – Traffic signals/signs, white/coloured lines, different road surface/pacing, and presence of a median and/or grass strip. We separated out *Bicycle lanes and quality* (presence/absence and quality); 2) ***Aesthetics/Beatification*** – natural features, man-made landscaping, street trees, street furniture/public art; 3) ***Community disorder*** - presence of litter/garbage, vacant/derelict buildings, buildings poorly maintained; 4) **Urbanization/density** – street density, vehicle density, density of parked cars; and 5) ***Overall appeal*** – assessed using a sum of four Likert scales by the central observers indicating level of agreement for each of the statements ‘neighbourhood is safe/pedestrian friendly,’ ‘streets and sidewalks in the neighbourhood are suitable for walking’, ‘buildings, homes and structures in this neightbourhood are very attractive’ and ‘neighbourhood as a whole is aesthetically appealing’.

**Table 5:** Summary scores: Method to summarise scores based on combining constructs in similar domains.

Domain (min/max possible)	Items and points allocated to scale
Urban density (0/9)	Street density (0–3)
	Vehicle density (0–3)
	Parked cars (0–3)
Aesthetics/Beautification (0/12)	Natural features (1 if yes to open field/body of water/mountain/hill/green belt forest/desert, 0 otherwise)
	Street trees (0–3)
	Man-made landscaping (0–3)
	Street furniture (benches, trash cans, bus shelters, street lamps) (1 if yes to bench/trash can/bus shelter/street lamp, 0 otherwise to maximum of 4)
	Public art (0–1)
Community disorder (0/4)	Litter/garbage present (0–1)
	Graffiti present (0–1)
	Derelict buildings (0–1)
	Buildings poorly maintained (0–1)
Community appeal (4/16)	‘Neighbourhood is safe/pedestrian friendly’ (1 to 4)
	‘Streets and sidewalks in the neighbourhood are suitable for walking' (1 to 4)
	'Buildings, homes and structures in this neighbourhood are very attractive' (1 to 4)
	‘Neighbourhood as a whole is aesthetically appealing (1 to 4)’
Pedestrian safety (0/14)	Sidewalks, (0–1)
	Sidewalk completeness (0–2)
	Sidewalk quality (0–3)
	Cross walks (0–1),
	Safety features of cross walks including white/colour painted lines, different road surface and paving (1 for each feature, 0 otherwise to maximum of 4),
	Traffic signals/signs (0–1)
	Median and/or grass strip (0–2)
Bike lanes and quality (range 0 to 4)	Present (0–1)
	Quality (0–2)

As indicated above, the scores were calculated by summing items in each domain, giving roughly equal weighting to each feature. When creating these scores we chose to exclude some items that we had collected in the evaluation tool. We excluded the following items as they did not fit well with existing constructs for all countries studied: Q27 - presence of buildings, Q28 – number of buildings, Q29 - awnings, Q8 - parking lots, Q9- street width. Further, we excluded Q13 – number and types of vehicles, as there were few data points and some of the measures were unreliable or irrelevant for some regions within countries. We excluded the following items because they had poor or borderline measures of reliability, few counts, fitted more than one domain or were interpreted differently in different settings, these were: Q11 obstacles to pedestrian walking, Q32 - building design. We separated bike lanes from the pedestrian safety domain due to few counts in some regions. We also removed the item on bike lanes from the ‘overall appeal’ domain as it was not reliable and there were few counts of bike lanes.

### Summary scores results

**Figure 2:**
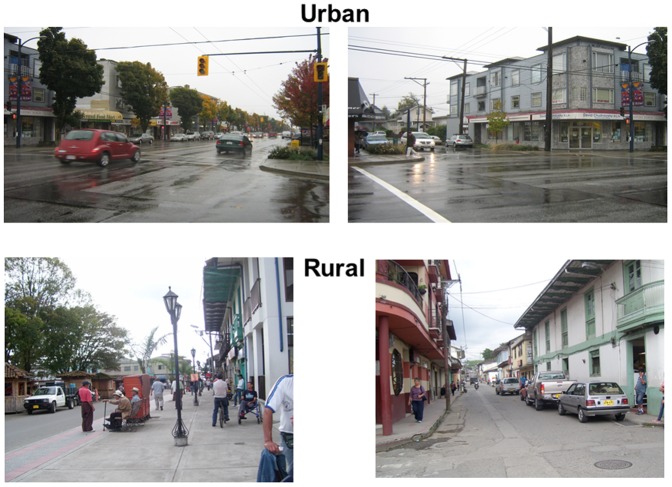
Examples of high scoring communities. In these communities from urban Canada and rural Colombia the common high-scoring characteristics are complete sidewalks, several planted trees, traffic signals, and pedestrian traffic signs, well maintained buildings and roads and the presence of street furniture such as benches and street lamps.

**Figure 3:**
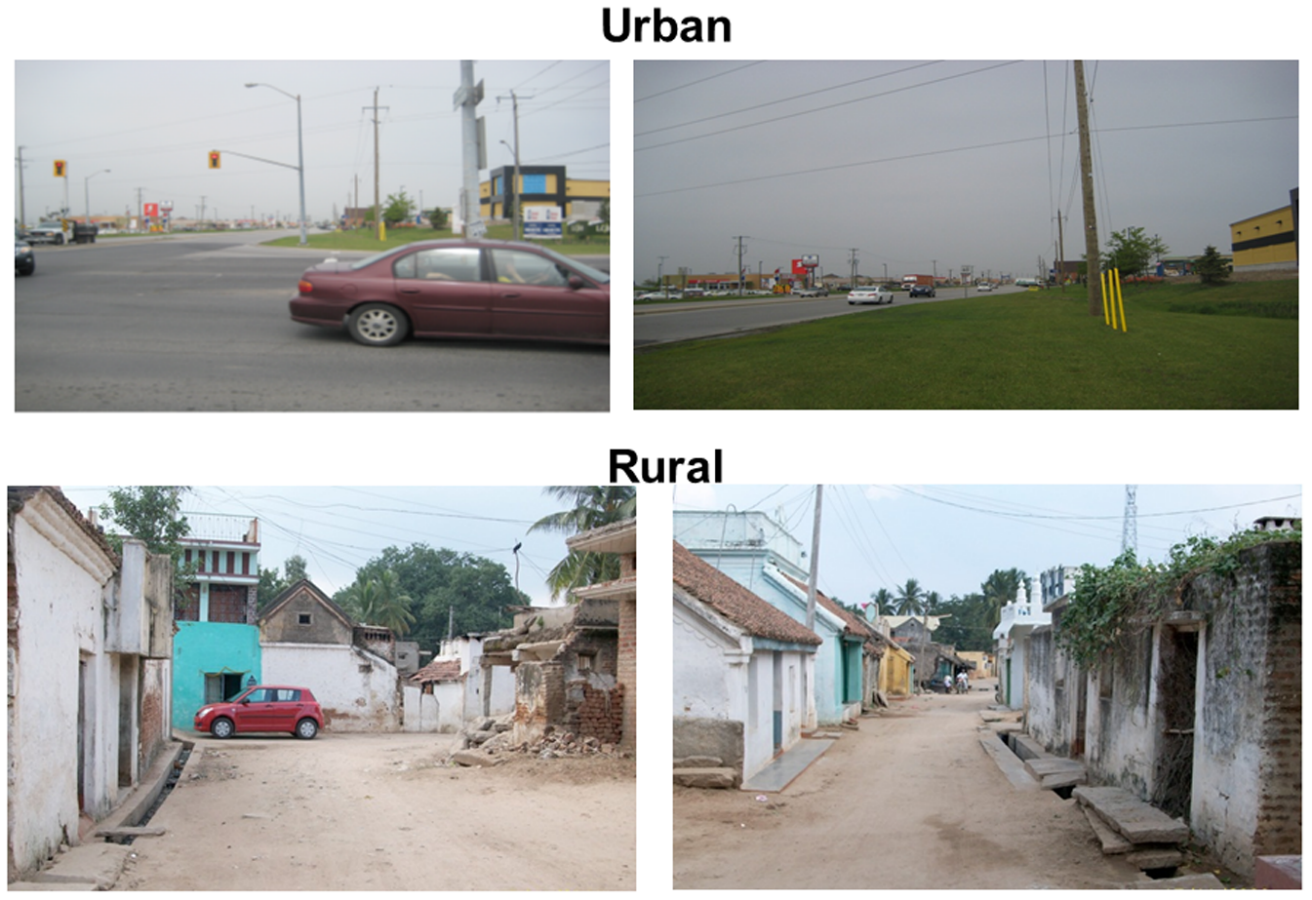
Examples of low-scoring communities. These are pictures of 2 low scoring communities overall. You can see in communities from urban Canada and rural India there is partial or no sidewalks, not many crosswalks, not many planted trees or aesthetically pleasing features. In addition the buildings are not well maintained.

The mean of 3 observers' summary scores for each domain are tabulated overall and by geographical region in **Table 6**. In **Table 7**, the mean scores for all communities are given across the three observers. The urbanization/density score and community disorder score were higher in Brazil/Colombia and the aesthetics/beatification score and pedestrian safety scores were higher in Canada. A sample of photos is shown in **Figure 2** and **Figure 3** for communities that scored high or low on multiple domains from Canada, Colombia and India.

**Table 6:** Mean Summary scores overall and by region and urban/rural.

	Country/community type (n)											
	Overall (86)		Canada (37)		Brazil/Colombia (20)		India/China (29)		Urban (50)		Rural (36)	
Domain Scale (range min/max)	Mean	SD	Mean	SD	Mean	SD	Mean	SD	Mean	SD	Mean	SD
Urban density (3/9)	6.09	1.27	6.13	1.11	6.53	1.33	5.74	1.36	6.30	1.11	5.80	1.44
Aesthetics/Beautification (0.7/10.3)	4.68	2.41	6.20	2.04	3.57	2.09	3.51	2.00	5.53	2.12	3.50	2.32
Community disorder (0/3)	1.10	0.79	0.71	0.57	1.17	0.59	1.55	0.92	1.01	0.64	1.23	0.96
Community appeal (4.3/15.7)	10.10	2.82	11.95	2.08	9.70	2.32	8.02	2.41	11.06	2.53	8.77	2.69
Pedestrian Safety (1/13.7)	7.89	4.20	11.18	2.22	6.30	2.68	4.79	4.03	9.46	3.34	5.71	4.33
Bike lanes and quality (0/4)	0.18	0.75	0.06	0.38	0.00	0.00	0.45	1.18	0.27	0.93	0.06	0.33

Figures in brackets are the range of each score determined from the distribution of mean scores for each summary score.

**Table 7:** Overall summary scores calculated by observer.

	Observer					
	1		2		3	
Domain Scale (range min/max)	Mean	SD	Mean	SD	Mean	SD
Urban density (3/9)	6.09	1.49	5.98	1.31	6.20	1.32
Aesthetics/Beautification (0/11)	4.56	2.51	4.93	2.51	4.55	2.46
Community disorder (0/3)	1.15	0.86	1.09	0.90	1.06	0.82
Community appeal (4/16)	10.36	3.31	10.08	3.08	9.86	2.60
Pedestrian Safety (1/14)	8.15	4.35	8.12	4.43	7.41	3.90
Bike lanes and quality (0/4)	0.19	0.77	0.17	0.74	0.17	0.74

Figures in brackets are the range of each score determined from the distribution of scores from all 3 observers.

## Discussion

This report describes a novel method to evaluate the built environment of communities by means of a set of photos taken according to a standardized method and applying a standard data extraction form to analyse each photo set for features of the community's physical environment. This method of community assessment is relatively rapid and involves minimal training of the on-site assessor (photographer). This could be important for studies that are conducted across multiple countries, particularly where there is not yet research expertise with regards to environmental assessment. The high reliability of the majority of measures indicates that measurement analysis is feasible and repeatable. The EPOCH photo instrument complements our previously reported instruments developed to collect environmental data relevant to cardiovascular health in large scale international studies - EPOCH 1 and EPOCH 2. EPOCH 1 is an objective environmental audit tool, in which a trained researcher directly observes and systematically records physical aspects of the environment and includes 5 sections, 1) community characteristics – a checklist of infrastructure and services, 2) Community observation walk – this walk starts at the ‘Start point’ described in this paper and is where the photos are taken 3) tobacco store assessment, 4) grocery store assessment and 5) local restaurant assessment. EPOCH 2 is a questionnaire to capture what participants observe in the community, their awareness of laws, regulations and their opinions about behaviours and laws.

Our findings have face validity because comparisons of measures and summary scores across regions fit directionally with expected constructs. The pedestrian facilities and safety characteristics observed were higher in the urban compared to the rural communities of Canada and there were relatively fewer pedestrian facilities in communities in India, which matches with expectations of individuals (many of the authors of this paper) that have travelled and observed both settings. While higher pedestrian and safety characteristics have been related to increased physical activity, [19] it is hard to ascertain whether the higher pedestrian facility and safety characteristics in Canada compared to other countries is more a function of the construct used to gain an objective measure of these characteristics being derived in countries with similar built environments such as the United States, Canada and Australia. [20] [21] It will require further investigation as to whether similar constructs about the built environment relate to physical activity in diverse countries. Similarly the higher measure of ‘overall appeal’ in Canada may be due to this measure being dominated by characteristics considered to be appealing in countries more similar to Canada or the assessment through a ‘westernised’ lens. The utility of the overall appeal measures also need to be examined in analyses of predictive validity. Our analyses here did find that some variables requiring the observer to express an opinion had high reliability. However in the case of judgments about suitability for biking there were specific inter-observer differences reflecting their own experiences. Our findings with respect to the three items with low ICCs described at the end of the results, has led us to remove the first and third of these and to clarify the definition for the second.

The study has some limitations: It was conducted in a convenience sample of communities in a limited number of countries, although compared with other studies to evaluate environmental assessment tools, this was an unusually diverse sample of communities. The photos were assessed only on constructs that have been previously reported to be associated with physical activity but the majority of these instruments have been developed in the United States. Consequently, there may be other aspects of walkability we did not capture. However, we intend to store the photos indefinitely which will permit re-analyses for additional domains when these are suggested by other research. The summary scores were created simply with equal or near equal weight put on each characteristic within domains and may require refinement in examining their predictive validity against outcomes such as physical activity. Finally, photos were only taken of commercial streets, unlike other built environment scales which included coverage of residential and commercial streets.

In conclusion, we describe an approach to environmental assessment that is relatively rapid, low-cost and simple with respect to the data collected, thus offering a means of obtaining data on the built environment as experienced by communities in diverse locations. Our team and our collaborators have now used this method to collect and analyse photographic data on the built environments from a large number of communities in the Prospective Urban Rural Epidemiology (PURE) study (conducted in 17 high, middle and low income countries) [18] and the Health In Times of Transition (HITT) study conducted across former Soviet Union countries. [22]

## Supporting Information

Appendix S1 **EPNET Instrument.**
(PDF)

Appendix S2 **Additional tables.**
(XLS)

Appendix S3 **Instruction manual for evaluating communities using photos with EP-NET EPOCH Photos - Neighbourhood Evaluation Tool.**
(PDF)

## References

[1] Sallis JF, Bowles HR, Bauman A, Ainsworth BE, Bull FC, et al (2009) Neighborhood environments and physical activity among adults in 11 countries. Am J Prev Med 36: 484–490. 19460656 10.1016/j.amepre.2009.01.031

[2] Foster C, Hillsdon M, Jones A, Grundy C, Wilkinson P, et al (2009) Objective measures of the environment and physical activity—results of the environment and physical activity study in English adults. J Phys Act Health 6 Suppl 1: S70–80. 19998852 10.1123/jpah.6.s1.s70

[3] Cerin E, Conway TL, Cain KL, Kerr J, De Bourdeaudhuij I, et al (2013) Sharing good NEWS across the world: developing comparable scores across 12 countries for the Neighborhood Environment Walkability Scale (NEWS). BMC Public Health 13: 309. 23566032 10.1186/1471-2458-13-309 PMC3642009

[4] Day K, Boarnet M, Alfonzo M, Forsyth A (2006) The Irvine-Minnesota inventory to measure built environments: development. Am J Prev Med 30: 144–152. 16459213 10.1016/j.amepre.2005.09.017

[5] Pomerleau J, Knai C, Foster C, Rutter H, Darmon N, et al (2013) Measuring the food and built environments in urban centres: reliability and validity of the EURO-PREVOB Community Questionnaire. Public Health 127: 259–267. 23375367 10.1016/j.puhe.2012.12.025

[6] Schaefer-McDaniel N, Caughy MO, O'Campo P, Gearey W (2010) Examining methodological details of neighbourhood observations and the relationship to health: a literature review. Soc Sci Med 70: 277–292. 19883966 10.1016/j.socscimed.2009.10.018

[7] Schaefer-McDaniel N, Dunn JR, Minian N, Katz D (2010) Rethinking measurement of neighborhood in the context of health research. Soc Sci Med 71: 651–656. 20579796 10.1016/j.socscimed.2010.03.060

[8] Gasevic D, Vukmirovich I, Yusuf S, Teo K, Chow C, et al (2011) A direct assessment of "obesogenic" built environments: challenges and recommendations. J Environ Public Health 2011: 161574. 22174727 10.1155/2011/161574 PMC3228298

[9] Chow CK, Lock K, Madhavan M, Corsi DJ, Gilmore AB, et al (2010) Environmental Profile of a Community's Health (EPOCH): an instrument to measure environmental determinants of cardiovascular health in five countries. PLoS One 5: e14294. 21170320 10.1371/journal.pone.0014294 PMC3000812

[10] Corsi DJ, Subramanian SV, McKee M, Li W, Swaminathan S, et al (2012) Environmental Profile of a Community's Health (EPOCH): an ecometric assessment of measures of the community environment based on individual perception. PLoS One 7: e44410. 22973446 10.1371/journal.pone.0044410 PMC3433440

[11] Wang C, Burris MA (1997) Photovoice: concept, methodology, and use for participatory needs assessment. Health Educ Behav 24: 369–387. 9158980 10.1177/109019819702400309

[12] Charreire H, Mackenbach JD, Ouasti M, Lakerveld J, Compernolle S, et al (2014) Using remote sensing to define environmental characteristics related to physical activity and dietary behaviours: a systematic review (the SPOTLIGHT project). Health Place 25: 1–9. 24211730 10.1016/j.healthplace.2013.09.017

[13] Chow CK, Lock K, Teo K, Subramanian SV, McKee M, et al (2009) Environmental and societal influences acting on cardiovascular risk factors and disease at a population level: a review. Int J Epidemiol 38: 1580–1594. 19261658 10.1093/ije/dyn258 PMC2786248

[14] Cerin E, Saelens BE, Sallis JF, Frank LD (2006) Neighborhood Environment Walkability Scale: validity and development of a short form. Med Sci Sports Exerc 38: 1682–1691. 16960531 10.1249/01.mss.0000227639.83607.4d

[15] Frank LD, Sallis JF, Saelens BE, Leary L, Cain K, et al (2010) The development of a walkability index: application to the Neighborhood Quality of Life Study. Br J Sports Med 44: 924–933. 19406732 10.1136/bjsm.2009.058701

[16] Pikora TJ, Bull FC, Jamrozik K, Knuiman M, Giles-Corti B, et al (2002) Developing a reliable audit instrument to measure the physical environment for physical activity. Am J Prev Med 23: 187–194. 12350451 10.1016/s0749-3797(02)00498-1

[17] Weich S, Burton E, Blanchard M, Prince M, Sproston K, et al (2001) Measuring the built environment: validity of a site survey instrument for use in urban settings. Health Place 7: 283–292. 11682328 10.1016/s1353-8292(01)00019-3

[18] Teo K, Chow CK, Vaz M, Rangarajan S, Yusuf S (2009) The Prospective Urban Rural Epidemiology (PURE) study: examining the impact of societal influences on chronic noncommunicable diseases in low-, middle-, and high-income countries. Am Heart J 158: 1–7 e1.. 19540385 10.1016/j.ahj.2009.04.019

[19] Lovasi GS, Schwartz-Soicher O, Neckerman KM, Konty K, Kerker B, et al (2013) Aesthetic amenities and safety hazards associated with walking and bicycling for transportation in New York City. Ann Behav Med 45 Suppl 1: S76–85. 23011913 10.1007/s12160-012-9416-z PMC3632298

[20] Parsons JA, Singh G, Scott AN, Nisenbaum R, Balasubramaniam P, et al (2010) Standardized observation of neighbourhood disorder: does it work in Canada? Int J Health Geogr 9: 6. 20146821 10.1186/1476-072X-9-6 PMC2831024

[21] Foster S, Giles-Corti B (2008) The built environment, neighborhood crime and constrained physical activity: an exploration of inconsistent findings. Prev Med 47: 241–251. 18499242 10.1016/j.ypmed.2008.03.017

[22] Watson K, Roberts B, Chow C, Goryakin Y, Rotman D, et al (2013) Micro- and meso-level influences on obesity in the former Soviet Union: a multi-level analysis. Eur J Public Health 23: 291–298. 22645239 10.1093/eurpub/cks054

