# Supplementary material for: A Novel Method to Evaluate the Community Built Environment Using Photographs – Environmental Profile of a Community Health (EPOCH) Photo Neighbourhood Evaluation Tool
Source: PLoS One. 2014 Nov 4;9(11):e110042. doi: 10.1371/journal.pone.0110042 (PMC4219679; doi:10.1371/journal.pone.0110042)
Supplement: Appendix S1 — EPNET Instrument. (PDF) [file pone.0110042.s001.pdf]

EPOCH Photos-Neighbourhood Evaluation Tool (EP-NET)

Country Name: **Centre #**:

Community Name: **EPOCH ID**:

Date of photo taken: **No. of Photos taken:**

(YYYY/MM/DD)

Places for Walking and Biking

1. **Are there any sidewalks present in the photos?**

   ☐ No ☐ Yes

2. **Indicate what type of sidewalks are present in the photos: (Mark only one)**

   ☐ Partial sidewalks (sidewalks are only on one/some sides of the street)

   ☐ Complete sidewalks (sidewalks present on all sides of street)

3. **What type of material is used to construct most of the sidewalks present in the photos? (Mark only one)**

   ☐ Concrete ☐ Gravel

   ☐ Paving bricks ☐ Grass

   ☐ Asphalt ☐ Sand

4. **How would you rate the overall quality of the sidewalks present in the photos? (Mark only one)**

   ☐ Sidewalks are undergoing repair

   ☐ Sidewalks are poorly maintained

   ☐ Somewhat maintained

   ☐ Very well maintained

5. **Are bicycle lanes present in the photos?**

   ☐ No ☐ Yes

EPOCH Photos-Neighbourhood Evaluation Tool (EP-NET)

6. **Indicate the quality of the bicycle lanes seen in the photos: (Mark only one)**
   - ☐ Low quality
   - ☐ Medium quality
   - ☐ High quality
7. **Indicate whether grass/dirt strip is present between the sidewalk and the road: (Mark only one)**
   - ☐ None present
   - ☐ Present on one or some sides of the street
   - ☐ Present on all sides of the street

Streets in the Neighbourhood

8. **Indicate how many parking lots are present in the photos: (Mark only one)**
   - ☐ None present
   - ☐ 1 present
   - ☐ 2 present
   - ☐ $\geq$ 3 present
9. **Indicate the width of the streets/roads present in the photos: (Mark only one)**
   - ☐ 1 lane wide
   - ☐ 2 or 3 lanes wide
   - ☐ 4 or 5 lanes wide
   - ☐ > 5 lanes wide
10. **Indicate the level of pedestrian density seen in the photos (Mark only one)**
    - ☐ No pedestrians seen in photo
    - ☐ Low
    - ☐ Moderate
    - ☐ Heavy

EPOCH Photos-Neighbourhood Evaluation Tool (EP-NET)

11. **Indicate the number of obstacles seen in the photos: (Mark only one)**
    - ☐ None present
    - ☐ 1 or 2 present
    - ☐ Some present
    - ☐ Many present

12. **Indicate the level of moving (not parked) motor vehicle density seen in the photos: (Mark only one)**
    - ☐ No vehicles seen
    - ☐ Low
    - ☐ Moderate
    - ☐ Heavy

13. **Indicate the number of the following vehicles present on the streets of this neighbourhood.**

Bicycles:	___	Rickshaw:	____
Cars:	___	Motorcycles/Scooters:	____
Bus:	___	Trucks:	____

14. **Indicate the amount of parked cars present in photos: (Mark only one)**
    - ☐ None Present
    - ☐ 1 or 2 present
    - ☐ 3 or 4 present
    - ☐ Greater than 4 present

Safety from Traffic

15. **Are there crosswalks present?**
    - ☐ No ☐ Yes

EPOCH Photos-Neighbourhood Evaluation Tool (EP-NET)

16. **Indicate the number of crosswalks present with signs or markings in the photos: (Mark only one)**

    ☐ 1 or 2 present

    ☐ 3 or 4 present

    ☐ Greater than 4 present

17. **Do(es) the crosswalk(s) have any of the following?**

    N ☐ **Y** ☐ White/coloured painted lines

    N ☐ **Y** ☐ Different road surface and paving

    N ☐ **Y** ☐ Traffic signals

    N ☐ **Y** ☐ Stop/Yield signs

    N ☐ **Y** ☐ Pedestrian activated signal

    N ☐ **Y** ☐ Pedestrian crossing signs

18. **Indicate type of median strip present in the photos: (Mark only one)**

    ☐ No median strip present

    ☐ 1 or 2 pr

    ☐ 3 or 4 present

    ☐ Greater than 4 present

<u>**Neighbourhood Aesthetics**</u>

19. **Indicate if any of the following natural features are present in the photos: (Mark all that apply)**

    ☐ Open field

    ☐ Body of water (lakes, ponds, oceans, creeks, etc.)

    ☐ Mountains/hills

    ☐ Greenbelt/forest

    ☐ Desert

EPOCH Photos-Neighbourhood Evaluation Tool (EP-NET)

20. **What percentage of the photographic scenes is taken up by the natural features mentioned in question '19'? ___**

21. **Indicate the number of planted trees that line the streets (does not include natural forestry): (Mark only one)**

- ☐ None Present
- ☐ 1 or 2 Present
- ☐ Some Present
- ☐ Many Present

22. **Indicate the number of man-made landscapes present in the photos (includes plants, bushes, flower beds/pots, fountains, gardens, etc; excluding trees.)**

- ☐ None Present
- ☐ 1 or 2 present
- ☐ Some present
- ☐ Many Present

23. **Indicate the amount of graffiti present in the photos: (Mark only one)**

- ☐ None Present
- ☐ 1 or 2 present
- ☐ Some present
- ☐ Many present

24. **Indicate the amount of litter/garbage present in the photos: (Mark only one)**

- ☐ None Present
- ☐ 1 or 2 present
- ☐ Some Present
- ☐ Many Present

EPOCH Photos-Neighbourhood Evaluation Tool (EP-NET)

25. Indicate if any of the following types of street furniture(s) are present in the photos: (Mark all that apply)

- ☐ Benches
- ☐ Parking meters
- ☐ Trash Cans
- ☐ Street Lamps
- ☐ Newspaper boxes
- ☐ Bus Shelters
- ☐ Bike racks
- ☐ Phone Booths

26. Indicate the amount of public art displayed in the photos (may include murals, decorative signs, sculptures, etc.)

- ☐ None Present
- ☐ 1 or 2 Present
- ☐ Some Present
- ☐ Many Present

27. Are buildings or houses present in the photos?

☐ **No** ☐ **Yes**

28. Indicate the total number of buildings, houses, and/or structures seen in the photos: ___

29. Indicate the number of awnings present on buildings or homes: (Mark only one)

- ☐ None present
- ☐ 1 or 2 present
- ☐ Some present
- ☐ Many Present

30. Indicate the amount of derelict or vacant buildings and homes present in the photos: (Mark only one)

- ☐ None present
- ☐ 1 present
- ☐ 2 present
- ☐ $\geq$ 3 present

EPOCH Photos-Neighbourhood Evaluation Tool (EP-NET)

31. **Evaluate if the exterior and structure of the buildings and/or houses is maintained. (Look for presence of paint cracks, broken or unstable exterior (windows, doors, and roofs), and check to see if building or home is well maintained to safe standards; exclude vacant or derelict buildings)**

- ☐ Most houses and/or building are poorly maintained
- ☐ Some buildings and/or houses are well maintained and some are poorly maintained
- ☐ All buildings and/or houses are well maintained

32. **Evaluate the diversity of the design/architecture of buildings' and/or houses seen in the photos: (Mark only one)**

- ☐ All are similar in design/architecture
- ☐ Some range of design
- ☐ Many ranges of designs

<u>**Neighbourhood Satisfaction**</u>

Indicate the level of your agreement to the following statements: (Mark only one for each question)

33. **The neighbourhood is safe/pedestrian friendly**

- ☐ Strongly disagree
- ☐ Somewhat disagree
- ☐ Somewhat agree
- ☐ Strongly agree

34. **The streets and sidewalks in the neighbourhood are suitable for walking**

- ☐ Strongly disagree
- ☐ Somewhat disagree
- ☐ Somewhat agree
- ☐ Strongly agree

EPOCH Photos-Neighbourhood Evaluation Tool (EP-NET)

35. The streets and sidewalks in the neighbourhood are suitable for biking

- [ ] Strongly disagree
- [ ] Somewhat disagree
- [ ] Somewhat agree
- [ ] Strongly agree

36. The buildings, homes, and structures in this neighborhood are very attractive.

- [ ] Strongly disagree
- [ ] Somewhat disagree
- [ ] Somewhat agree
- [ ] Strongly agree

37. The neighbourhood as a whole is aesthetically appealing.

- [ ] Strongly disagree
- [ ] Somewhat disagree
- [ ] Somewhat agree
- [ ] Strongly agree

Name of Evaluator:

(first initial). (Last name)
