# Supplementary material for: A Novel Method to Evaluate the Community Built Environment Using Photographs – Environmental Profile of a Community Health (EPOCH) Photo Neighbourhood Evaluation Tool
Source: PLoS One. 2014 Nov 4;9(11):e110042. doi: 10.1371/journal.pone.0110042 (PMC4219679; doi:10.1371/journal.pone.0110042)
Supplement: Appendix S3 — Instruction manual for evaluating communities using photos with EP-NET EPOCH Photos - Neighbourhood Evaluation Tool. (PDF) [file pone.0110042.s003.pdf]

Instruction manual for evaluating communities using photos with

EP-NET

EPOCH Photos- Neighbourhood Evaluation Tool

Introduction

Studies show the characteristics of a community's built environment are related to risk factors for chronic disease such as physical activity and obesity. To enable investigation of the relationship between community built environments and disease risk factors including genetic factors requires the development of simple and practical methods that can be used in a variety of communities to quantify community characteristics. In EPOCH (Environmental Profile of a Community's Health) we are developing methods to evaluate communities for large-scale international studies. One method of measuring the physical features of community's built-environment is evaluating the neighborhood design qualities visually. EPOCH Photos-Neighborhood Evaluation Tool (EP-NET) is an instrument developed to evaluate sets of photographs taken within each community.

With this tool you will be evaluating the built environment and physical features of a neighbourhood. You will be using a set of a minimum of 4 photos taken at the start point during the EPOCH community assessment walk. The photographs taken are 360° views (back, front, right side, and left, side) from the start point and one photo taken from across the start point. You will be evaluating each item in the instrument using all the photos present in each set for each community.

Structure of the Tool

EP-NET assesses the built environment in five aspects; Places for Walking and Biking, Streets in the Neighbourhood, Safety from traffic, Neighbourhood Surroundings; Neighbourhood Satisfaction.

Places for Walking and Biking

Q1-Q2. Sidewalks are paths or walkways for pedestrians that are situated alongside a road/street.

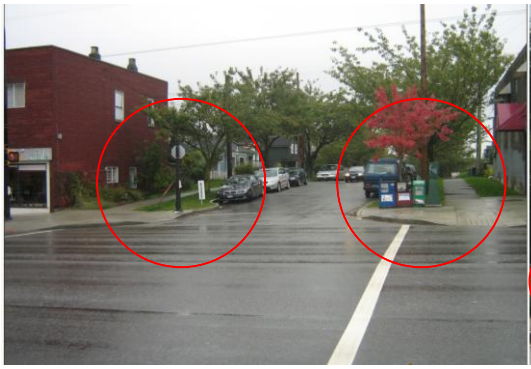

Complete Sidewalk: sidewalks are on all sides of streets

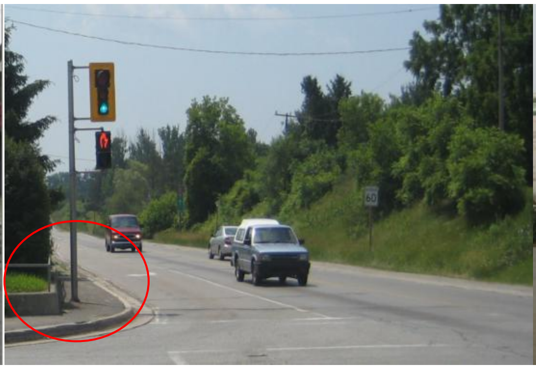

Partial Sidewalk: sidewalks are only on some or one side of the street

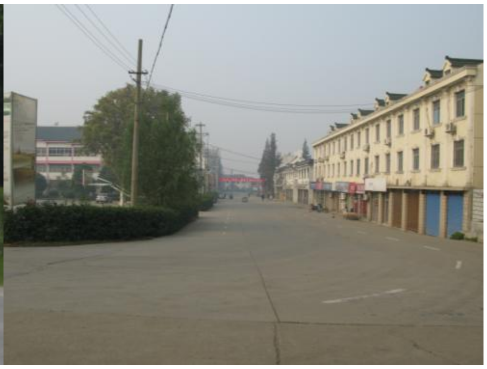

No Sidewalk present

Q3. Sidewalk Material:

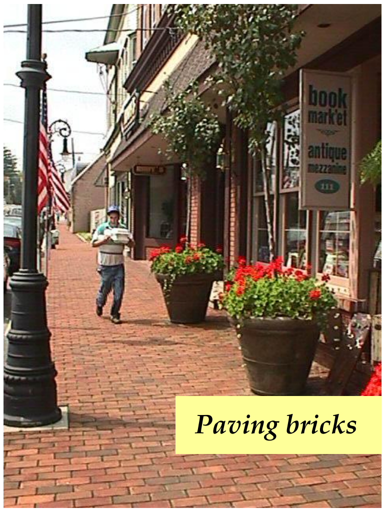

Paving bricks

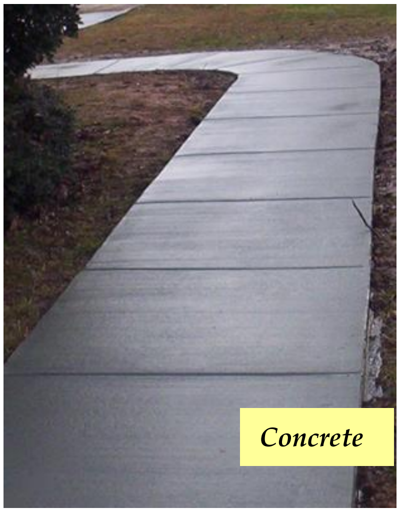

Concrete

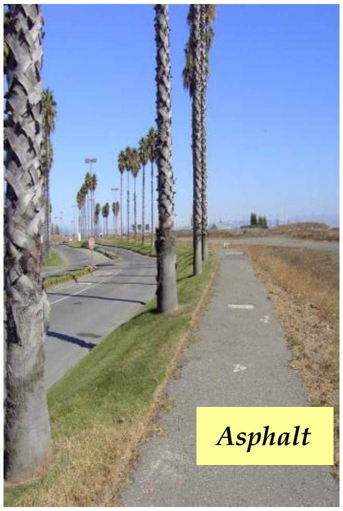

Asphalt

Q4. Sidewalk Maintenance: Assess how well the sidewalks are maintained—Look for any bumps. cracks, holes or weeds on the sidewalk that make it difficult for pedestrians to walk

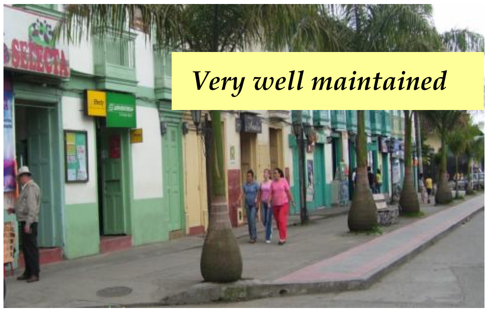

Very well maintained

Sidewalk has no bumps, cracks, holes and weeds

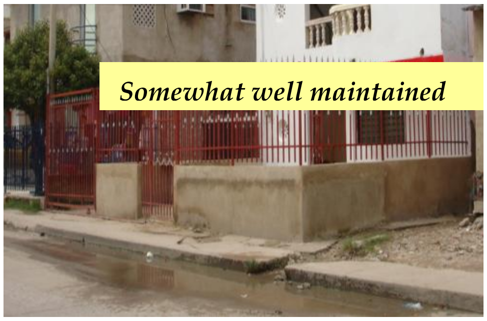

Somewhat well maintained

Sidewalk has few bumps, cracks, holes and weeds

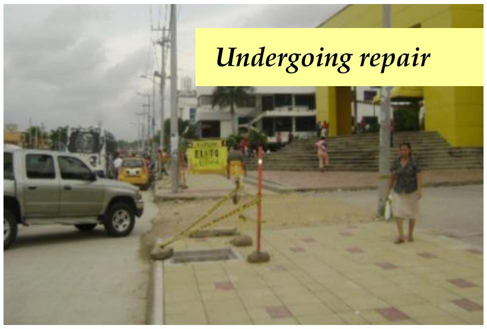

Undergoing repair

Sidewalk is temporarily unusable due to construction

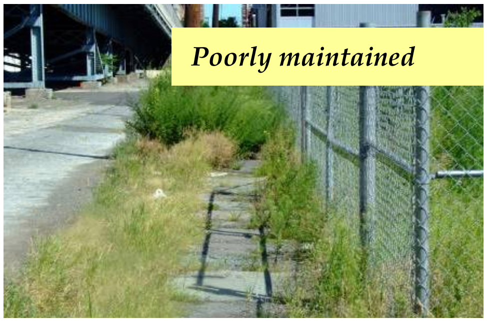

Poorly maintained

Sidewalk has many bumps, holes, cracks, weeds

Q6. Bicycle lanes are designated spaces on the roads/streets, which is solely for the use of cyclists

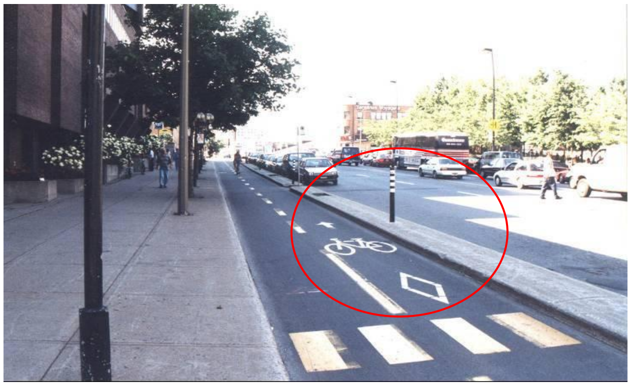

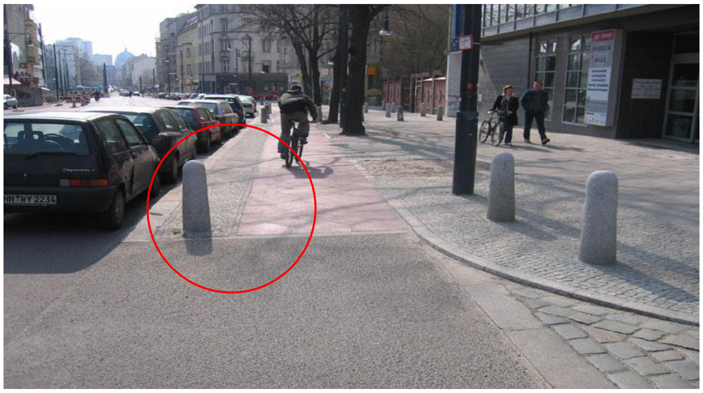

High quality bicycle lanes: have signs/labels, and have special markings, and are demarcated (e.g. raised curb) from traffic by a physical barrier.

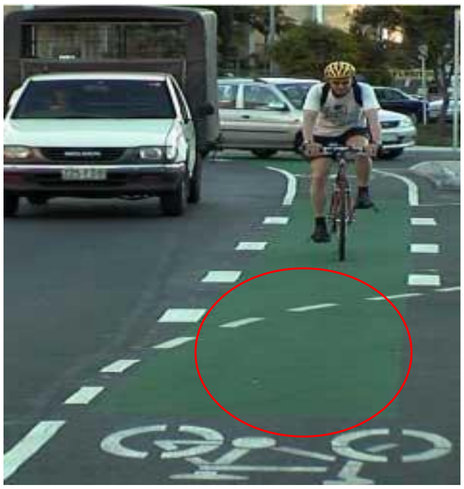

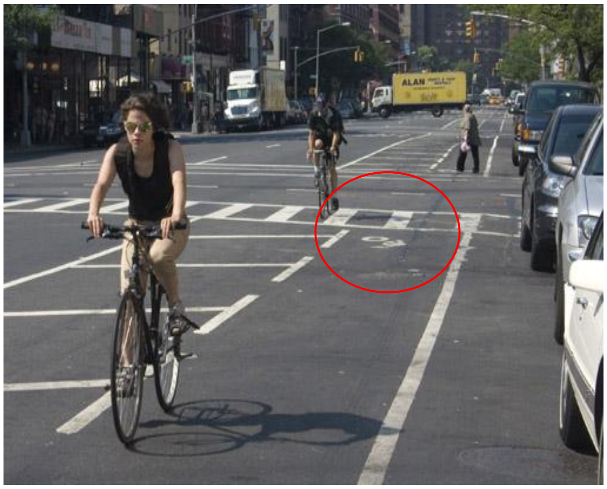

Medium quality bicycle lanes: have signs/labels, has special markings but is not demarcated

Low quality bicycle lanes: have signs/labels, does not have special markings and is not demarcated

Q7. Grass/dirt strip is present between the sidewalk and the road:

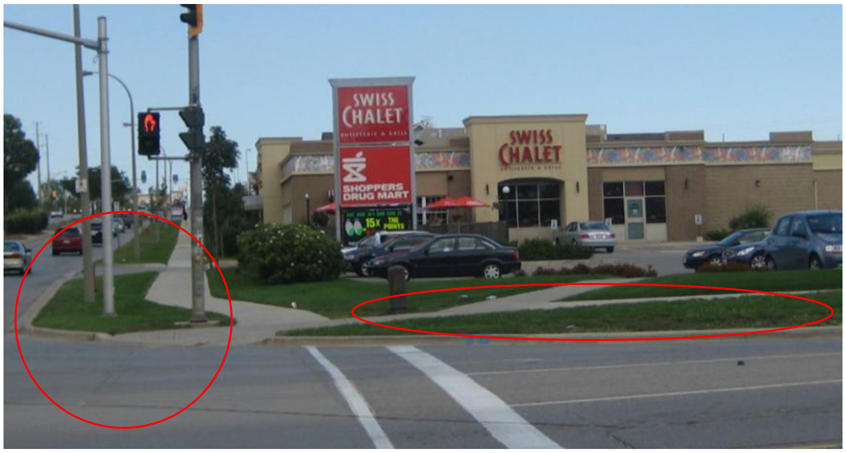

Streets in the Neighbourhood

Q8. Parking lots are cleared areas that is intended for parking vehicles

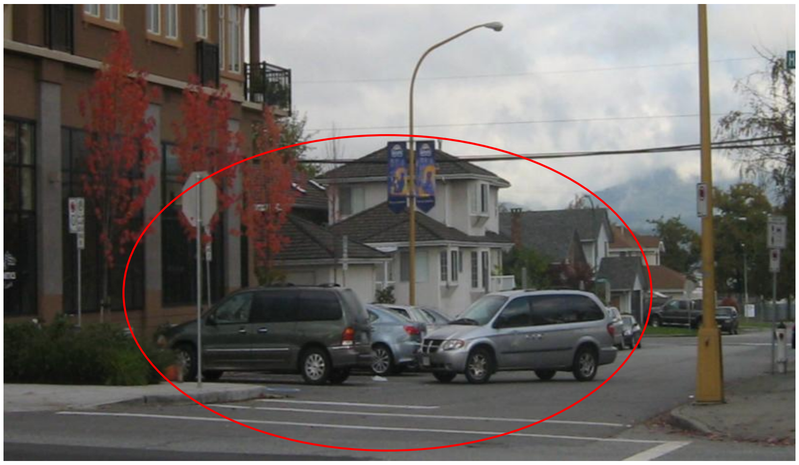

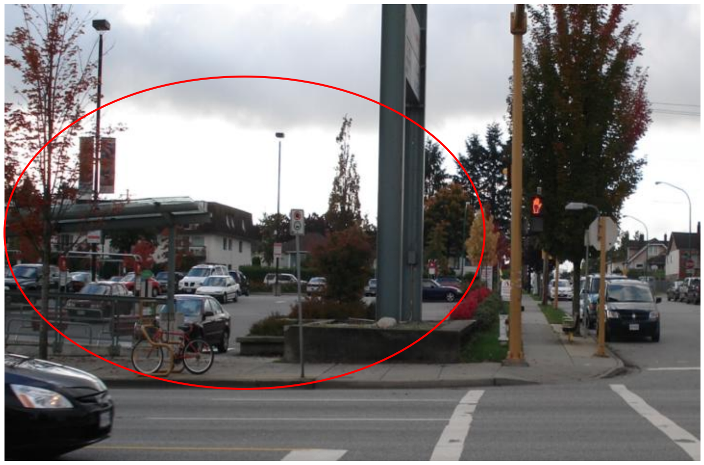

Q9. Width of streets/roads: the widths of roads or streets can be determined by counting the number of lanes present. Lanes are usually divided by painted lines or can be counted by the lines of vehicles present on the street.

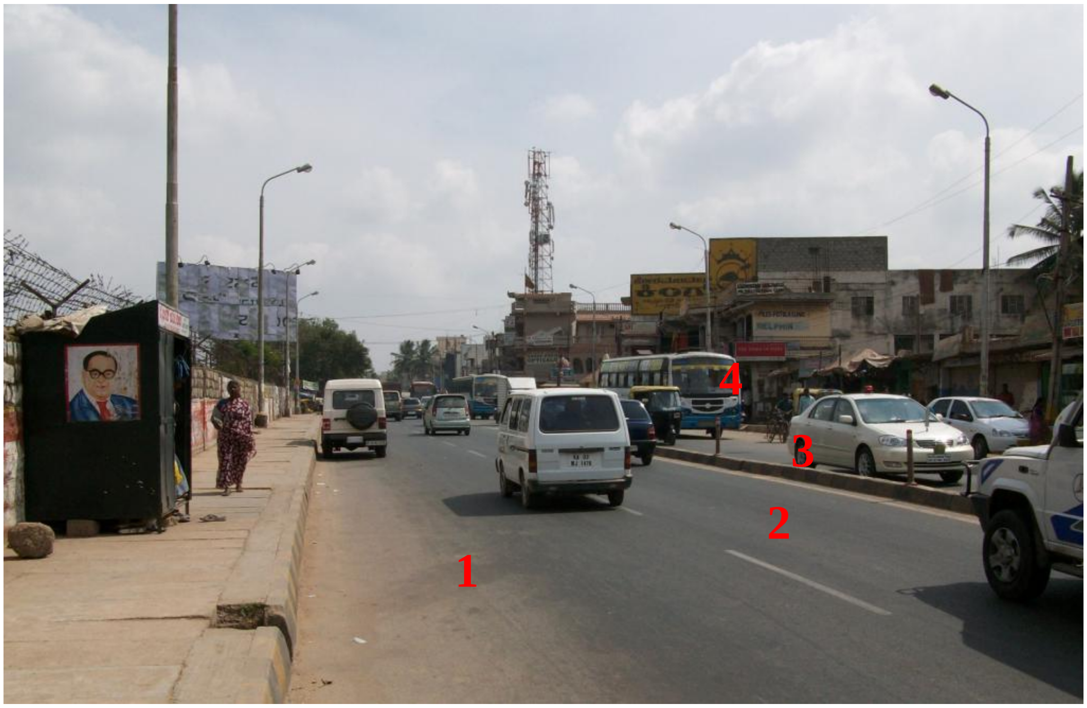

The picture above has 4 lanes in total.

Q10. Pedestrian density evaluates how crowded the streets are and how easy flowing the pedestrian traffic is.

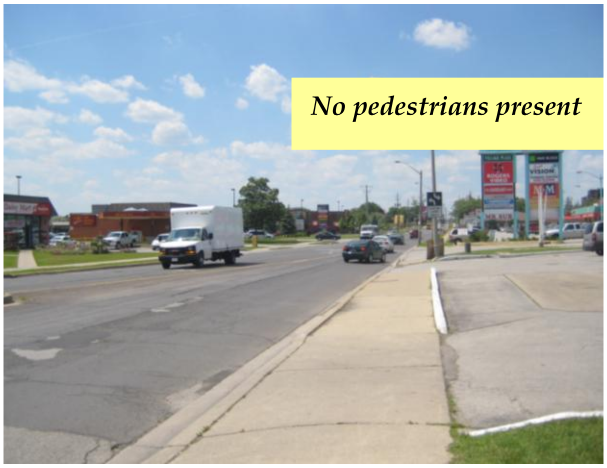

No person(s) seen on the streets

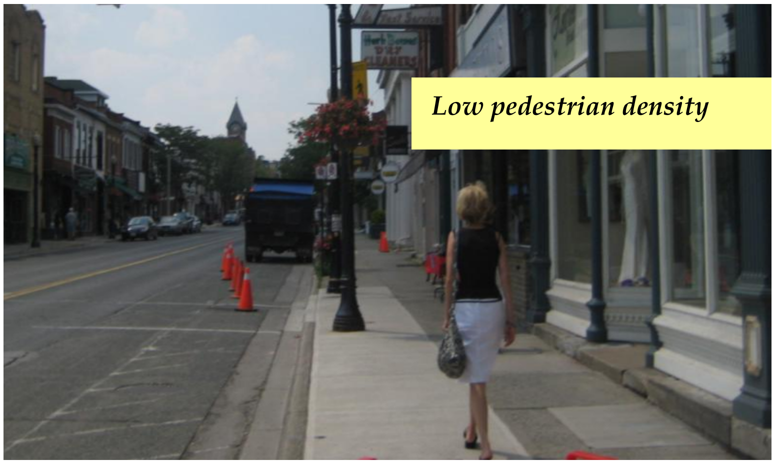

Not at all crowded, very few people (1 or 2) on street

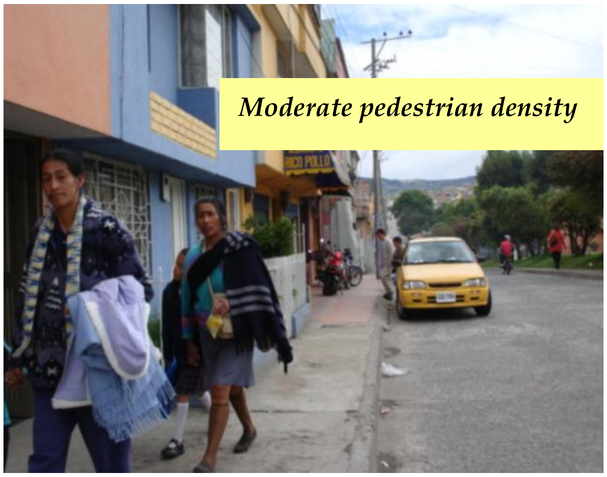

Streets are not very crowded & pedestrian traffic is easy flowing

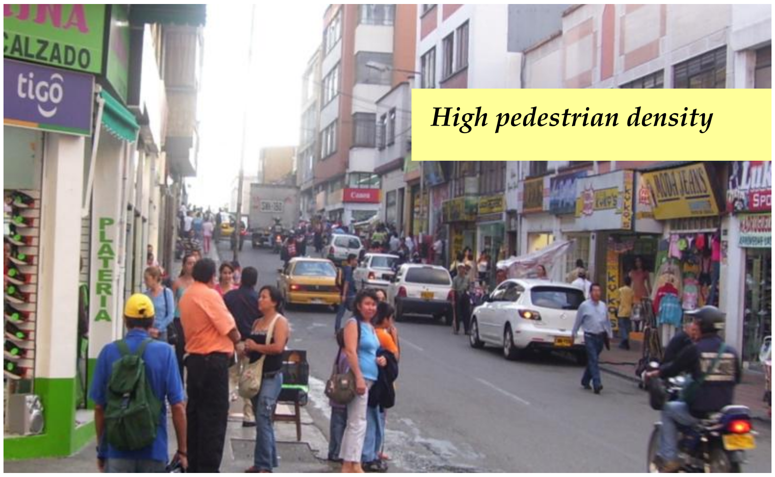

Streets are very crowded and pedestrian traffic is not easy flowing

Q11. Obstacles are any construction or hindrances present on streets/roads that may make it difficult for pedestrians to walk comfortably and safely.

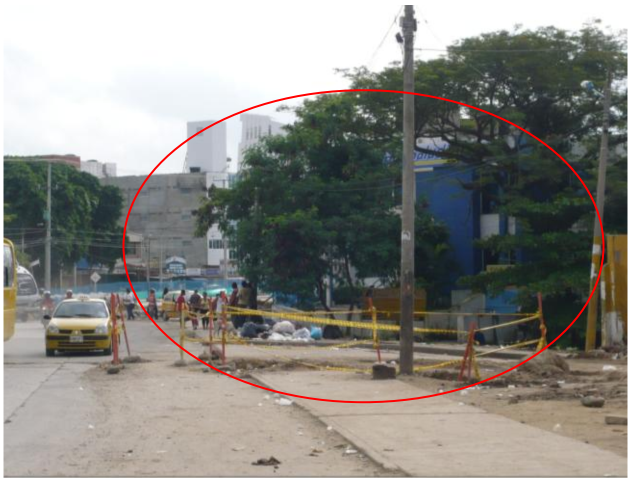

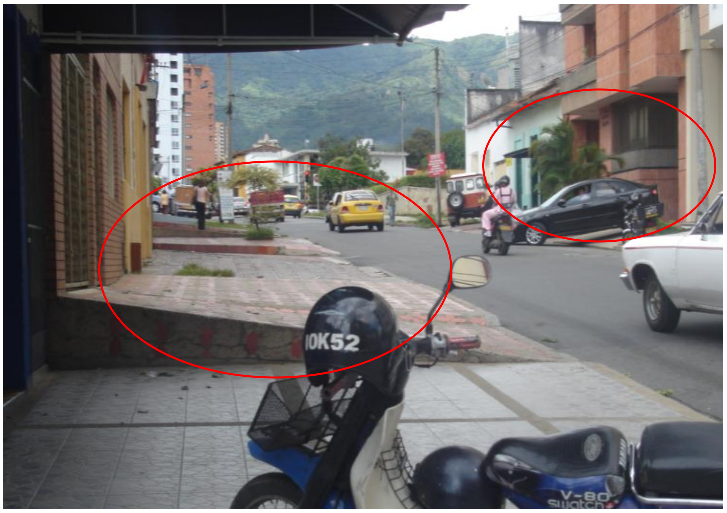

Obstacles can include construction on sidewalks, dangerous sidewalk ramps/bumps, cars or other vehicles blocking the sidewalk. If no sidewalks are present assess obstacles on roads that may hinder or make it difficult for walking.

Q12. Motor vehicle density: Evaluate the amount of un-parked motor vehicles present on the roads. Motor vehicles include cars, buses, taxis, trucks, motorbikes or anything that has an engine. **Un-parked** vehicles are moving and on the main roads and not parked on side of roads away from moving traffic.

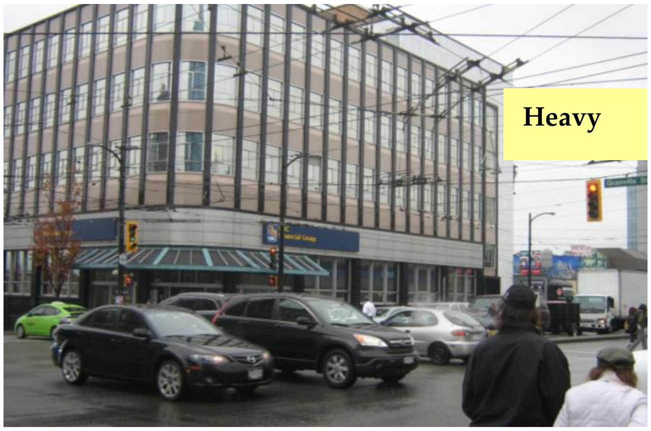

The roads seen are occupied by many motor vehicles, heavy traffic

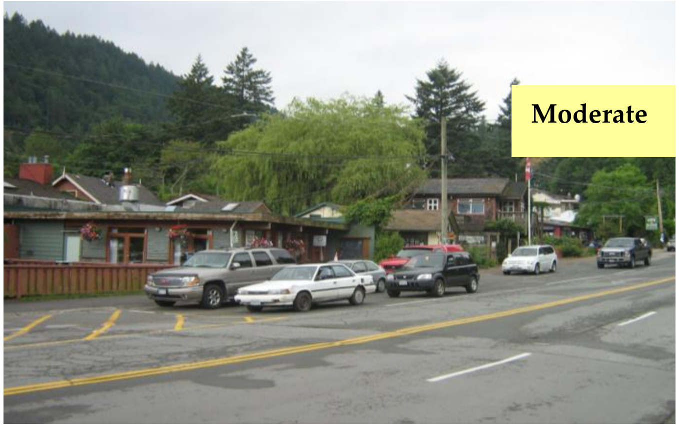

The roads seen are occupied by some motor vehicles, medium level of traffic

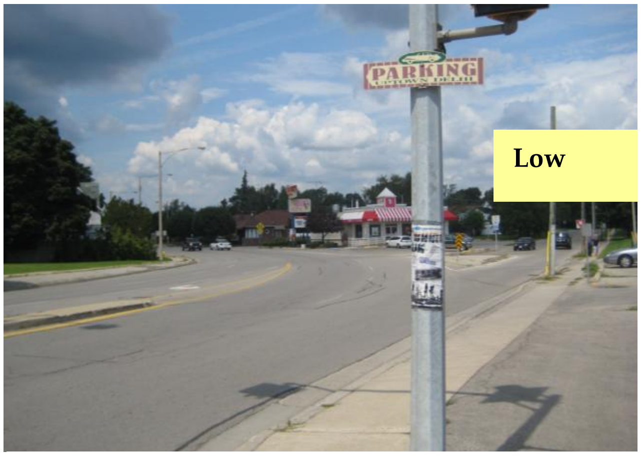

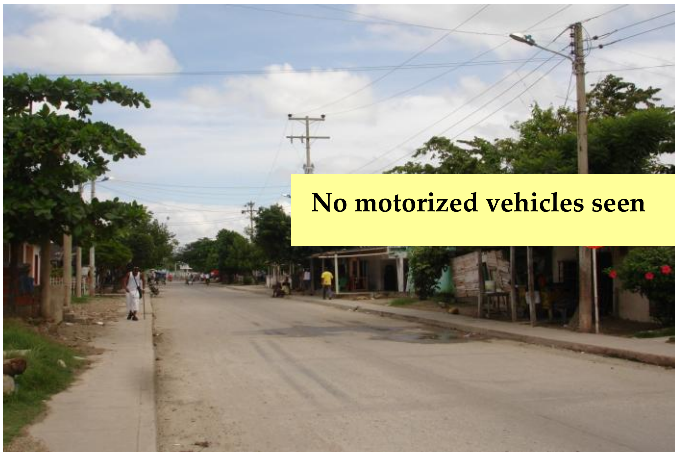

1 or 2 vehicles seen on roads, no traffic

Q13. Example of Vehicles:

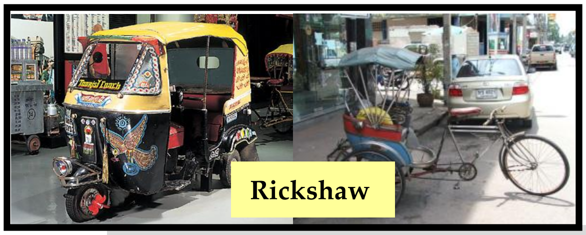

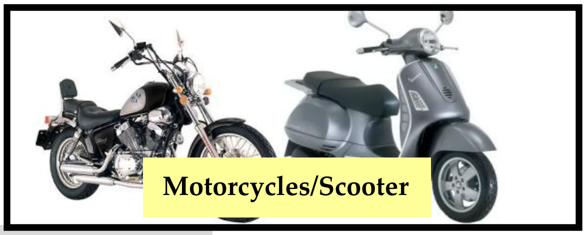

- *If one vehicle is seen in more than one picture, count as '1'*

Q14. Parked vehicles are vehicles that are away from traffic and are parked either in a designated lot or on sides if streets and/or houses.

Q15-Q16. Crosswalks are designated points on a road (usually at intersections), which are designed to help pedestrians cross safely.

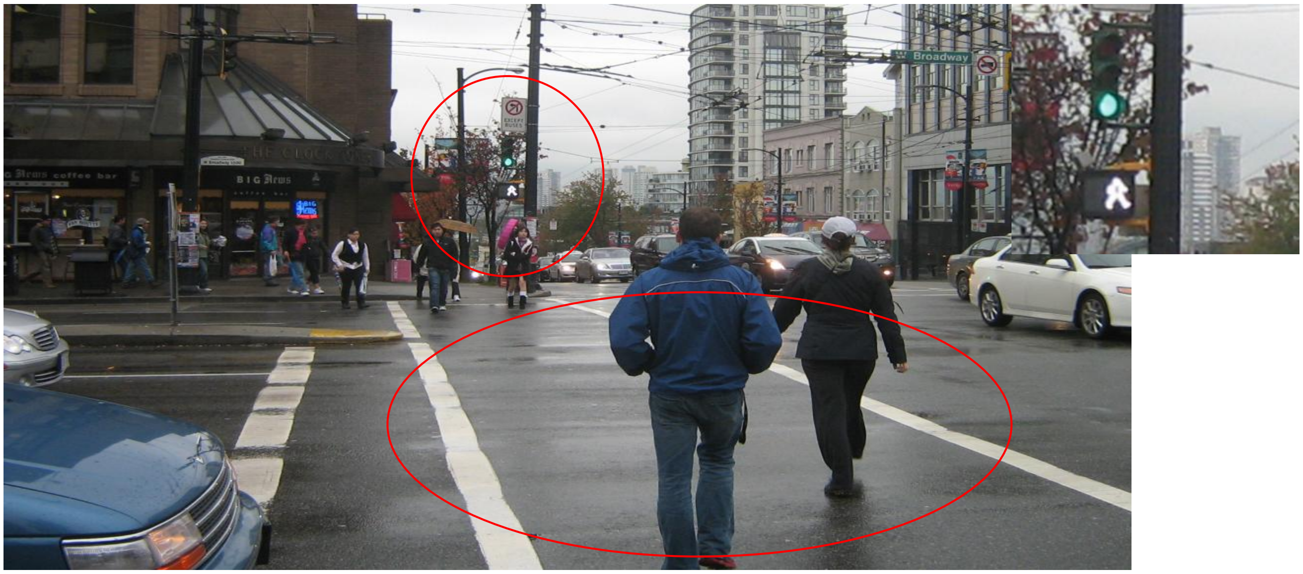

Q 17. *Crosswalks are usually identified by painted lines on the roads, pedestrian activated signals, or traffic signals that make motor vehiclists and pedestrians aware of on-going traffic. Crosswalks can also be marked by:*

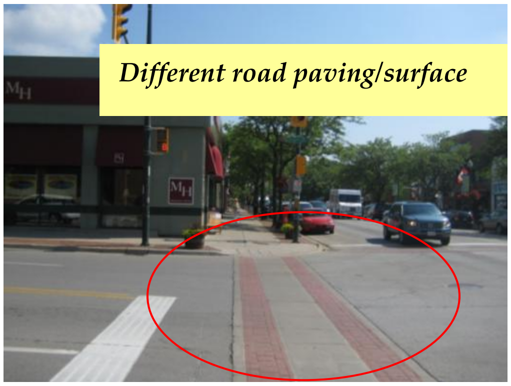

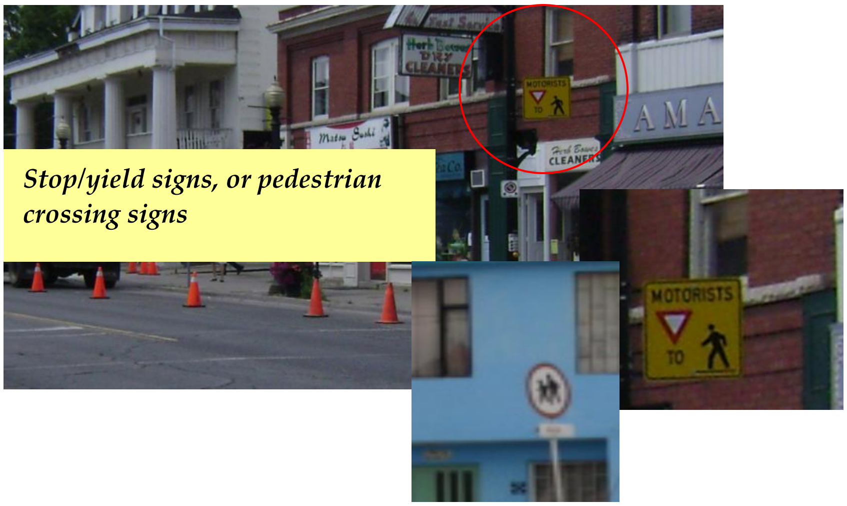

Q18. Median strips are bands located in the center of the road usually separating lanes down the middle of roads that are very wide. They are used for added safety for both on-going vehicle traffic and pedestrians.

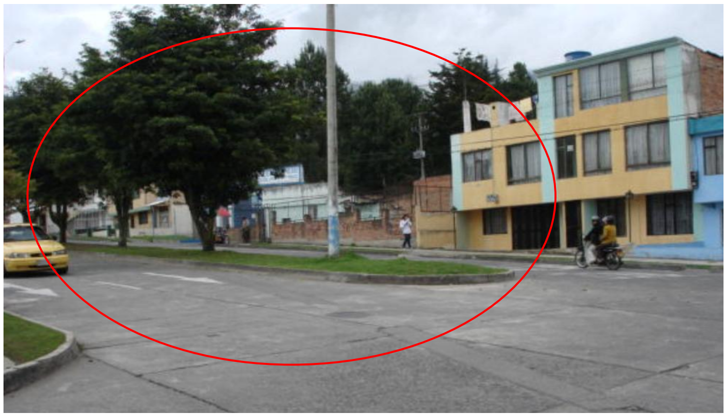

High quality median strip*: Have protruding slabs, which are specially decorated by planted trees and/or flowers and bushes*

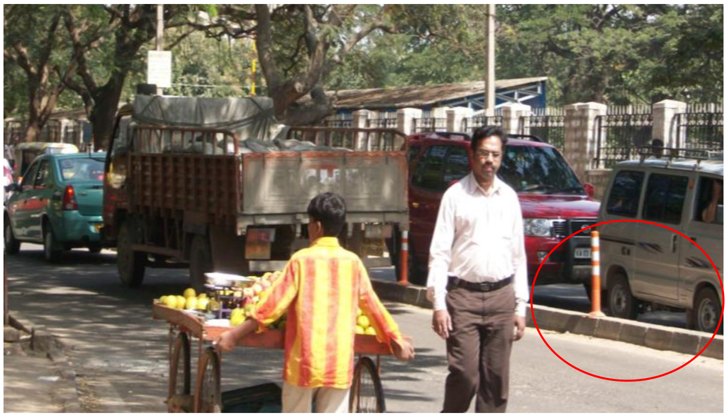

Median strip present*: A protruding slab is present but is not specially decorated*

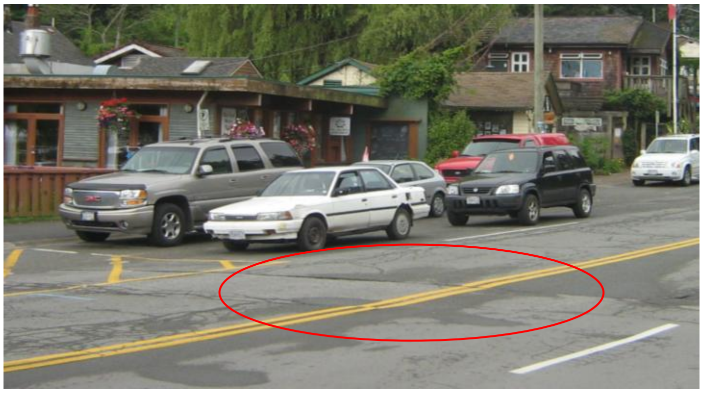

Low quality median strip*: No protrusion, only marked by coloured painted line.*

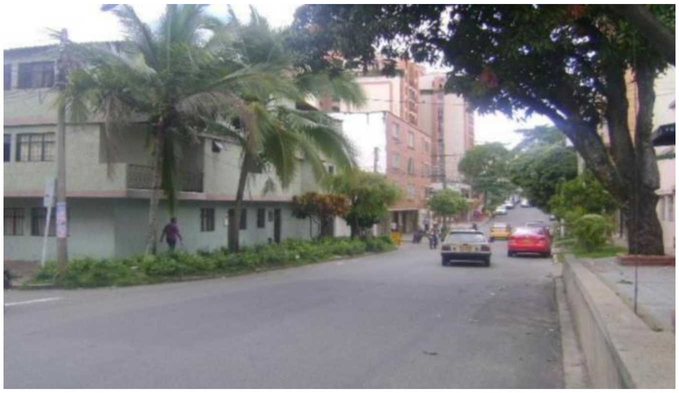

No median strip present

Neighbourhood Surroundings

Q19-Q20. Natural features include sceneries that are not man-made but present naturally. These can include open fields, bodies of water, mountains or hills, greenbelt/forests and deserts.

To evaluate the percentage of photographic scenes taken up by the natural features, estimate the average of how much of the natural feature is present in the entire set of photos being evaluated.

Example:
Natural feature present: Greenbelt/forest

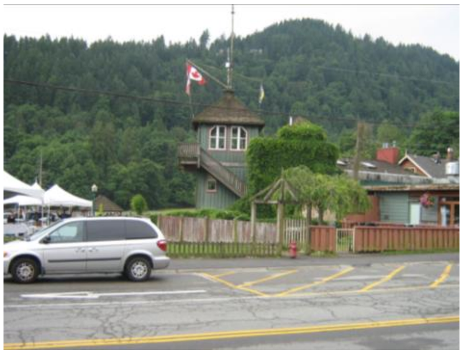

Photo 1: *~50% of photo taken up by natural feature*

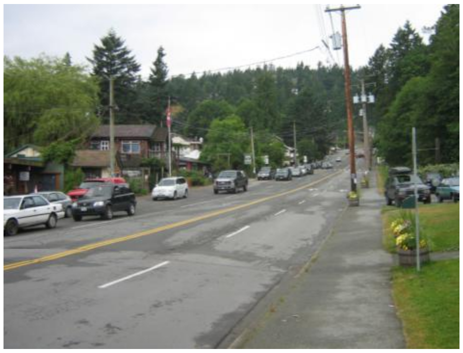

Photo 2: *~30% of photo taken up by natural feature*

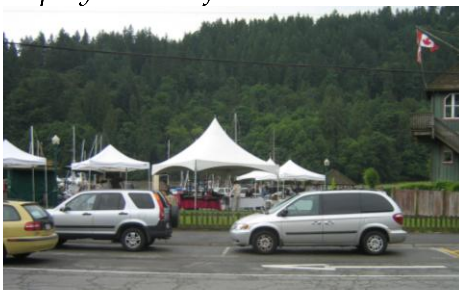

Photo 3: ~50% of photo taken up by natural feature

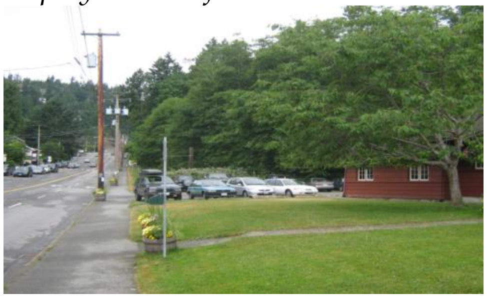

Photo 4: *~10% of photo taken up by natural feature*

Average percentage of scenes in 4 photos taken up by natural feature: ~ 35%

Q21. Trees planted: evaluate how many trees have been planted (not present naturally) along the streets and sidewalks in the neighbourhood

Q22. Man-made landscapes include plants, bushes, flower beds/pots, fountains, gardens or any features added to make the neighbourhood look aesthetically pleasing. This excludes trees.

Examples:

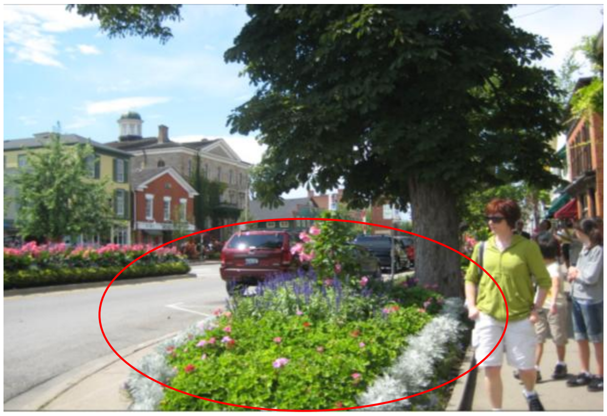

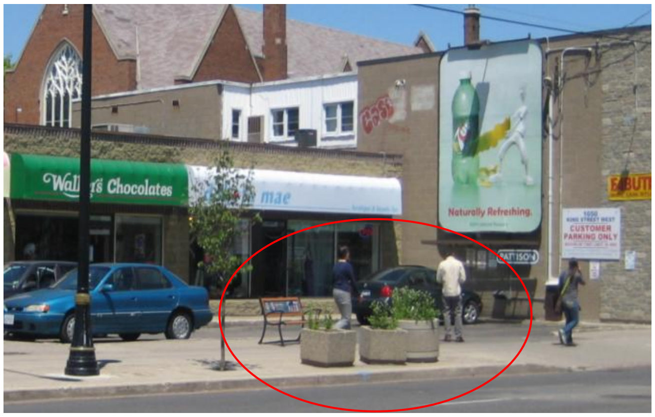

Q23. Graffiti are any images or words painted or marked in any manner on property.

Examples:

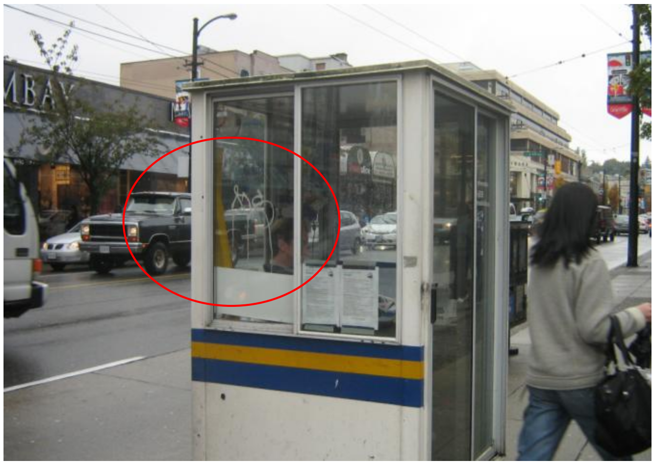

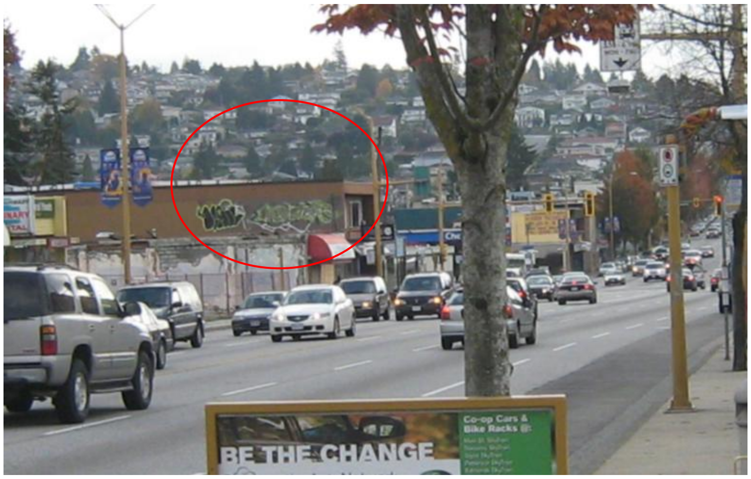

Q24. Litter/garbage is any waste disposed on the roads or sidewalks.

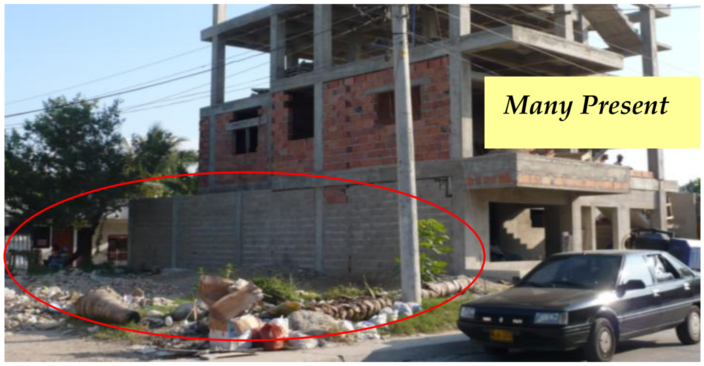

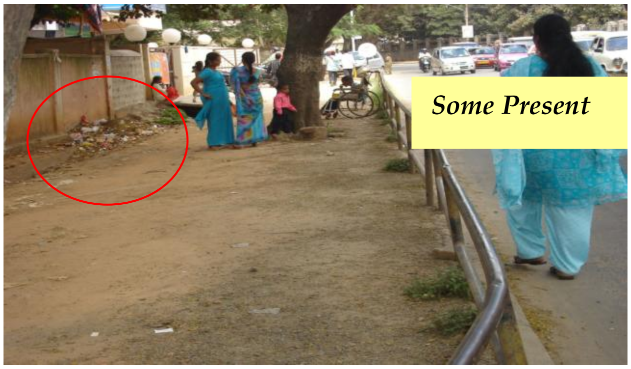

Q26. Awnings are overhangs that are attached to the exterior of buildings. They are used to give protection from sun, rain, or for aesthetic purposes.

Examples:

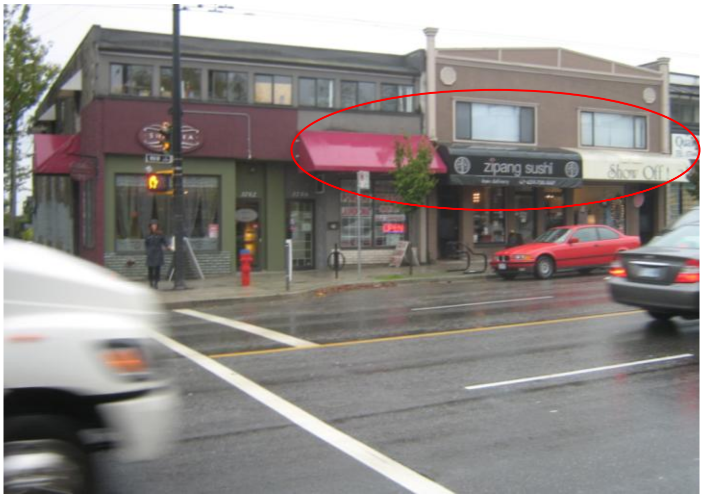

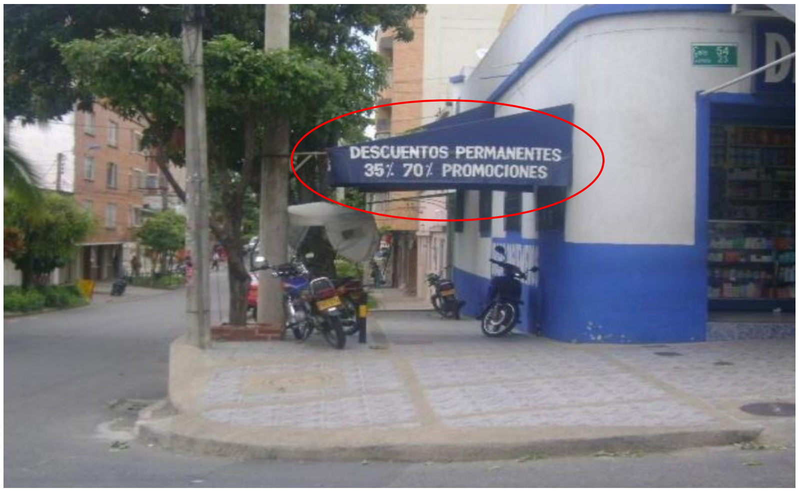

Q27. Public art includes artwork or murals, decorative signs, sculptures or any other items to give a neighbourhood added beauty.

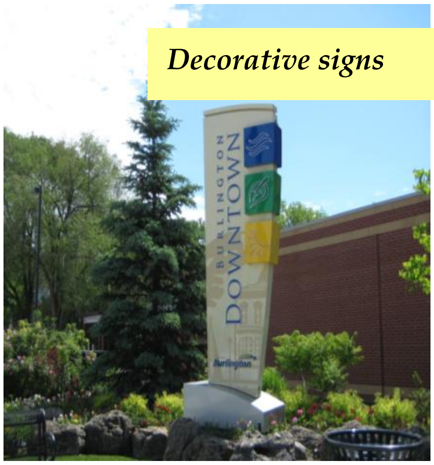

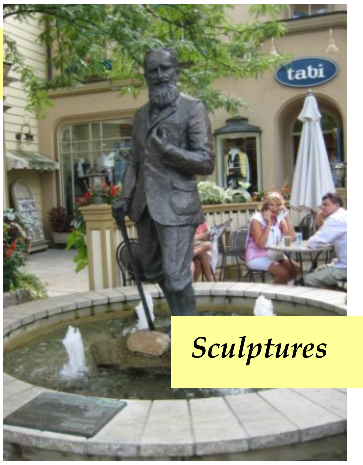

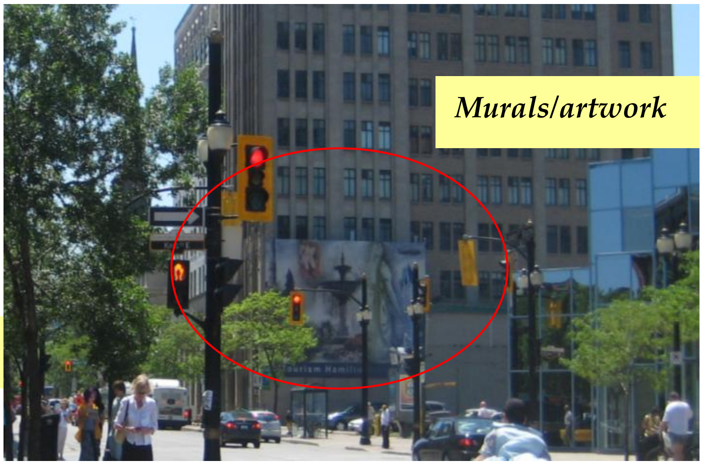

Q28. Number of buildings: Count how many buildings and/or houses are visible in each photo. If the buildings and/or houses repeat in other photos, count it only once. If several structures are attached, count as one.

Example-

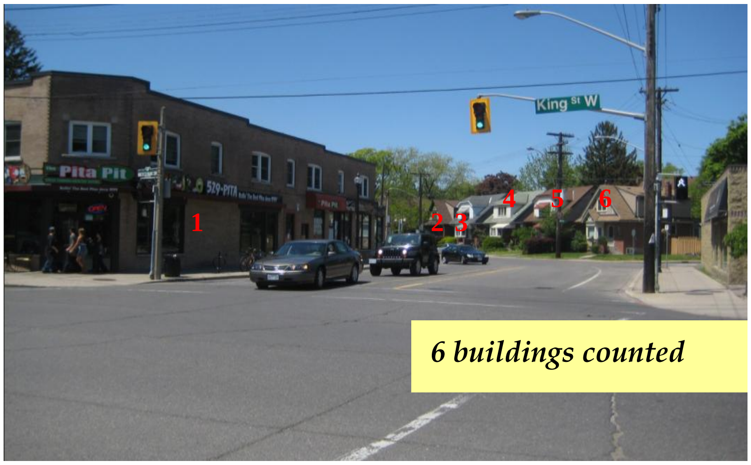

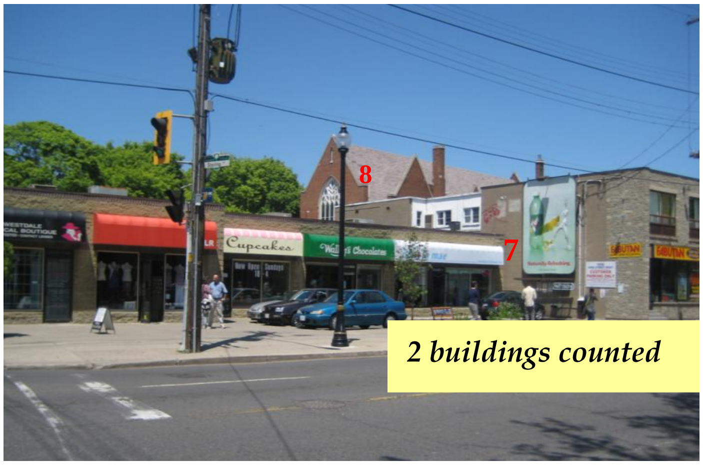

The above photo set has 8 buildings in total.

Q 29. Vacant buildings: Count how many derelict or vacant buildings are seen in the photo. This includes any buildings and/or houses that seem abandoned or have no human occupancy in them. Can also include buildings that are under-going construction.

Q31. Building maintenance: Evaluate the exterior and structure of buildings and/or houses. Look for presence of paint cracks, broken or unstable exterior such as in windows, doors, and roofs. Check to see if buildings and homes are well maintained to safe standards.

Q32. Overall impression of buildings' architecture: Evaluate building and/or houses' structures vary in architectural design or have the same design. If all buildings and or houses seen in the photos have different architecture then mark 'many ranges of design'. If some buildings and/or houses are different and some are similar then mark 'some range of design'. If there is no architectural or design differences present, then mark 'all are similar in design/architecture'.

Choosing between 'Some' and 'Many'

Several questions have the option 'some' and 'many'. When evaluating, choose 'some' if the item being evaluated is present in less than 60% of all photos seen. Choose 'many' if the item is in 60% or more of all the photos seen.

Neighbourhood Satisfaction

The set of questions in this section asks your level of agreement to statements pertaining to the overall walking and biking infrastructure of neighbourhood, the safety of pedestrians in the neighbourhood, the attractiveness of buildings and homes in the neighbourhood, and the overall aesthetic appeal of the neighbourhood.
